# The E3 ligase c-Cbl modulates microglial phenotypes and contributes to Parkinson’s disease pathology

**DOI:** 10.1038/s41420-025-02482-0

**Published:** 2025-04-17

**Authors:** Shumin Deng, Zhiyuan Zhang, Lu Liu, Chen Xu, Di Zhang, Lin Dong, Chunyan Gao, Xiaomin Wang, Zheng Fan

**Affiliations:** 1 Department of Pharmacology, School of Basic Medical Sciences, Capital Medical University, Beijing, PR China; 2 Department of Neurobiology, School of Basic Medical Sciences, Capital Medical University, Beijing, PR China; 3 Department of Pathology, National Cancer Center/National Clinical Research Center for Cancer/Cancer Hospital, Chinese Academy of Medical Sciences and Peking Union Medical College, Beijing, PR China; 4 Department of Clinical Medicine, Yanjing Medical College, Capital Medical University, Beijing, PR China; 5 Co-innovation Center of Neuroregeneration, Nantong University, Nantong, Jiangsu PR China

**Keywords:** Parkinson's disease, Microglia

## Abstract

Microglial activation, particularly the polarization between classical (M1 phenotype) and alternative (M2 phenotype) states, plays pivotal roles in the immune pathogenesis of Parkinson’s disease (PD), with the M1 phenotype exerting neurotoxic effects and the M2 phenotype conferring neuroprotection. Modulating microglial polarization toward the M2 phenotype holds therapeutic potential for PD. This study investigated the role of c-Cbl, an E3 ubiquitin ligase implicated in modulating microglial phenotypes and protecting dopaminergic neurons. Our findings revealed that c-Cbl^-/-^ mice exhibited motor deficits, reduced striatal dopamine levels, and progressive dopaminergic neuron loss in the substantia nigra (SN). Genetic ablation of c-Cbl significantly increased proinflammatory cytokine release and microglial activation in the SN, accompanied by a phenotypic shift from M2 to M1 polarization. Furthermore, stereotaxic c-Cbl knockdown in the SN exacerbated behavioral impairments and accelerated dopaminergic neuron degeneration in the MPTP-induced mouse model of PD. At the molecular level, c-Cbl deletion promoted M1 polarization of microglia through dysregulation of the PI3K/Akt signaling pathway, thereby impairing dopaminergic neuronal survival. Collectively, this study demonstrates that c-Cbl knockout recapitulates PD-like pathology and drives microglial activation. Our results establish that c-Cbl orchestrates the transition from neurotoxic M1 to neuroprotective M2 microglial phenotypes, highlighting its central role in PD immunopathogenesis. These findings suggest c-Cbl as a promising therapeutic target for modulating microglial polarization and alleviating PD symptoms.

## Introduction

Parkinson’s disease (PD) is recognized as the second most common age-related neurodegenerative disorder, affecting more than six million individuals worldwide [1]. The hallmark pathology of PD involves the gradual degeneration of dopaminergic neurons in the substantia nigra pars compacta (SNc) and the formation of α-synuclein-positive Lewy bodies within the remaining neurons [2, 3]. This degeneration of the nigrostriatal dopaminergic pathway results in the characteristic motor symptoms of PD, which include bradykinesia, muscle rigidity, resting tremor, and gait disturbances [4]. Among the various factors contributing to PD pathogenesis, microglia-mediated neuroinflammation is crucial, although traditional anti-inflammatory treatments have not shown substantial efficacy in clinical settings [5, 6].

Microglia are the resident immune cells of the central nervous system (CNS) and play a key role in regulating inflammation [7]. Microglial activation and subsequent inflammation-driven neurotoxicity critically contribute to dopaminergic neuron death in PD [8]. Depending on the degree of activation, the type of stimulation and the local factors present, microglial activation may evolve into different phenotypes: M1 and M2 [9]. In response to pathological stimuli, microglia can transition to a reactive state, known as the M1 phenotype, characterized by the secretion of proinflammatory mediators such as interleukin-1β (IL-1β), tumor necrosis factor-α (TNF-α), interleukin-6 (IL-6), and inducible nitric oxide synthase (iNOS). These factors are generally involved in defense mechanisms and pathogen clearance. On the other hand, M2 microglia, which represent the “alternative activation” state, possess anti-inflammatory properties that facilitate wound healing and tissue repair. The M2 phenotype is promoted by anti-inflammatory stimuli, including interleukin-4 (IL-4), arginase 1, CD206, and interleukin-10 (IL-10) [10, 11]. This phenotype is crucial for resolving chronic neuroinflammation, a component of PD and other neurodegenerative conditions [12]. Given that microglial phenotypes can shift during different stages of PD, modulating these transitions may augment their protective functions and have significant clinical therapeutic and research implications [13]. Consequently, a promising therapeutic strategy for PD involves curbing the detrimental M1 phase and fostering tissue homeostasis by either promoting M2 polarization or amplifying its beneficial effects [14]. The concept of manipulating microglial polarization from the M1 to the M2 phenotype as a means of treating PD has garnered significant attention.

Casitas B-lineage lymphoma (c-Cbl) is a RING finger E3 ubiquitin ligase encoded by a proto-oncogene and belongs to the Cbl protein family, which contains c-Cbl, Cbl-b and Cbl-c in mammals. It is well known for its role in the negative regulation of signaling pathways mediated by receptor tyrosine kinases [15, 16]. In the context of immune responses, c-Cbl has been shown to modulate inflammatory processes [17–21]. For instance, in dendritic cells, c-Cbl regulates dextran sodium sulfate-induced colitis by mediating the ubiquitination and degradation of the noncanonical NF-κB subunit RelB [21]. Our previous research indicated that c-Cbl is abundantly expressed in the CNS, particularly in the substantia nigra (SN), striatum, and hippocampus. We also demonstrated that microglial activation increases in the SNc of c-Cbl knockout mice following systemic administration of lipopolysaccharide (LPS). Furthermore, c-Cbl was found to inhibit the expression of LPS-induced proinflammatory cytokines and chemokines in microglia [17]. Despite these findings, the specific role of c-Cbl in PD pathogenesis has remained unclear.

In this study, we investigated the role of c-Cbl in PD by examining its effects in c-Cbl knockout mice. Our findings revealed that the absence of c-Cbl results in PD-like symptoms and enhances microglial activation in the SN, shifting microglial phenotypes from the anti-inflammatory M2 state to the proinflammatory M1 state. Moreover, we observed that c-Cbl knockdown in the SN exacerbates motor deficits and accelerates dopaminergic neurons degeneration in a 1-methyl-4-phenyl-1,2,3,6-tetrahydropyridine (MPTP) mouse model of PD. Mechanistically, we discovered that c-Cbl deletion facilitates the transition of microglia from the M2 phenotype to the M1 phenotype through the regulation of the PI3K/Akt signaling pathway, which subsequently impairs dopaminergic neuronal survival. These findings suggest that c-Cbl plays a crucial role in modulating microglial activation and neuronal survival in PD, making it a potential therapeutic target for this disease.

## Results

### Reduced c-Cbl expression in the substantia nigra of aged mice, MPTP-treated mice and PD patients

**Fig. 1:**
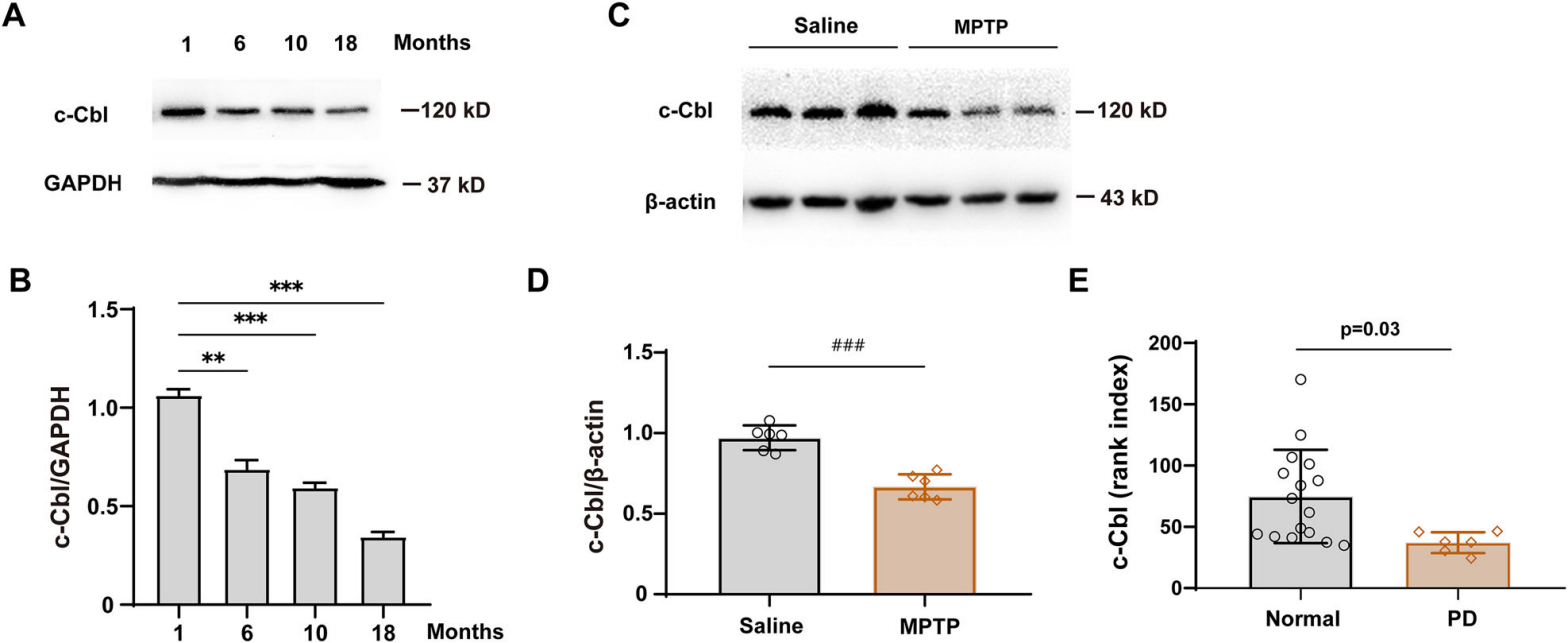
Reduced c-Cbl expression in the substantia nigra of aged mice, MPTP-treated mice and PD patients. **A** c-Cbl protein expression patterns in the SN of aged mice at different months. **B** Quantification of c-Cbl protein levels normalized to GAPDH in each group (*n* = 3). **C** c-Cbl protein expression in the SN of MPTP-treated mice versus saline-treated controls. **D** Quantification of c-Cbl protein levels normalized to β-actin (*n* = 6). **E** Bioinformatics analysis of c-Cbl mRNA levels in the SN of healthy controls (*n* = 6) and PD patients (*n* = 16) using the NCBI database (Reference Series: GSE7307). Data expressed as mean ± SEM; **p < 0.01, ***p < 0.001 vs. 1 month-old group. ^###^*p* < 0.001 vs. MPTP group.

As previously reported, c-Cbl is highly expressed in the CNS, particularly in the SN and striatum, which are associated with PD [17]. We assessed the protein expression of c-Cbl in the SN at various ages (1, 6, 10, and 18 months). The results showed a progressive decrease in c-Cbl expression with advancing age (Fig. 1A, B). To evaluate the role of c-Cbl in PD progression, we established a subacute PD mouse model using MPTP and measured c-Cbl expression in the SN. Compared with those in the saline group, the c-Cbl expression in the MPTP group was significantly lower (Fig. 1C, D). To determine whether c-Cbl was associated with PD development, we performed bioinformatics analysis of c-Cbl expression in patients with PD and healthy controls. In the GSE7307 dataset, the rank index of c-Cbl in patients with PD was substantially lower than that in control samples (Fig. 1E). Collectively, these findings suggest that c-Cbl is associated with aging and is potentially involved in PD progression.

### c-Cbl deficiency leads to motor dysfunction, reduced dopamine levels in the striatum, and loss of dopaminergic neurons in the substantia nigra

**Fig. 2:**
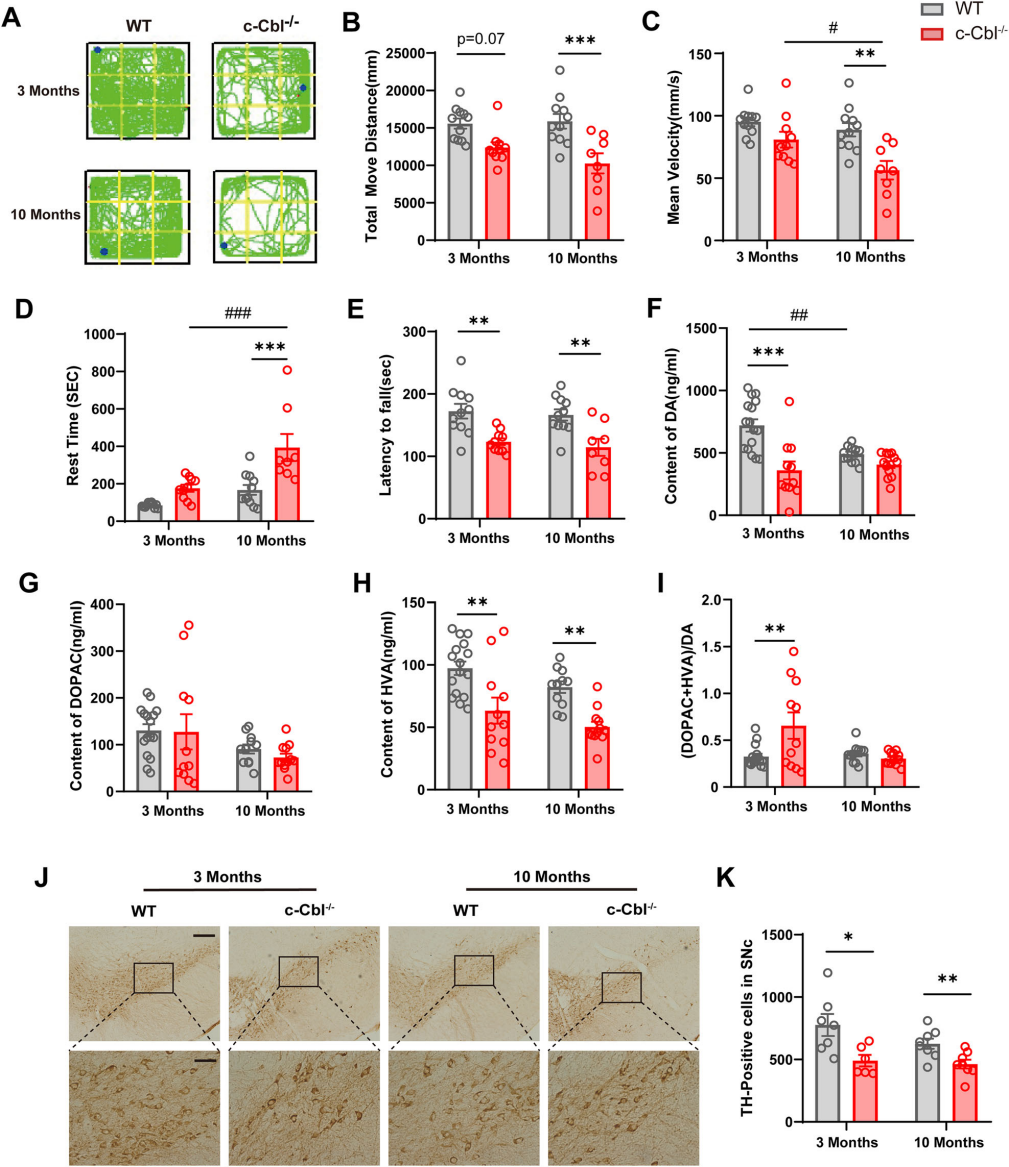
c-Cbl deficiency leads to motor dysfunction, reduced dopamine levels in the striatum, and loss of dopaminergic neurons in the substantia nigra. **A** Representative movement trajectories during open field tests. **B**–**D** Changes in locomotor behavior in 3 month-old and 10 month-old c-Cbl^-/-^ mice and wild-type (WT) littermates (*n* = 8–11): (**B**) total movement distance, (**C**) mean velocity, and (**D**) rest time were recorded in 30 min. **E** Changes in motor coordination and balance in 3 month-old and 10 month-old c-Cbl^-/-^ mice and WT mice. **F**–**H** The levels of DA (**F**) and its metabolites, DOPAC (**G**) and HVA (**H**), in the striatum were analyzed by HPLC (*n* = 11-16). **I** The ratio of (DOPAC + HVA)/DA was analyzed. **J** Microphotographs of TH-positive neurons in the SNc of 3-month-old and 10 month-old c-Cbl^-/-^ mice and WT mice. **K** Quantification of TH-positive neurons in the SNc (*n* = 6-8). Scale bars: 200 μm (above) or 50 μm (below). Data expressed as mean ± SEM; *p < 0.05, **p < 0.01, ***p < 0.001 vs. age-matched WT. ^#^*p* < 0.05, ^##^*p* < 0.01, ^###^*p* < 0.001 versus 3 month-old c-Cbl^-/-^ mice.

To investigate the role of c-Cbl in PD development, we used 3- and 10 month-old c-Cbl knockout (c-Cbl^-/-^) mice. Motor dysfunction was evaluated through voluntary movement behaviors. In the open field test, compared with WT mice, 10 month-old c-Cbl^-/-^ mice showed significantly reduced total movement distance and average speed, as well as increased resting time. Furthermore, 10-month-old c-Cbl^-/-^ mice exhibited more severe motor deficits than 3 month-old c-Cbl^-/-^ mice (Fig. 2A–D). In the rotarod test, both 3 month-old and 10 month-old c-Cbl^-/-^ mice spent significantly less time on the rotarod than did WT mice (Fig. 2E). These results suggest that c-Cbl knockout exacerbates motor impairment, especially in aged mice.

**Fig. 3:**
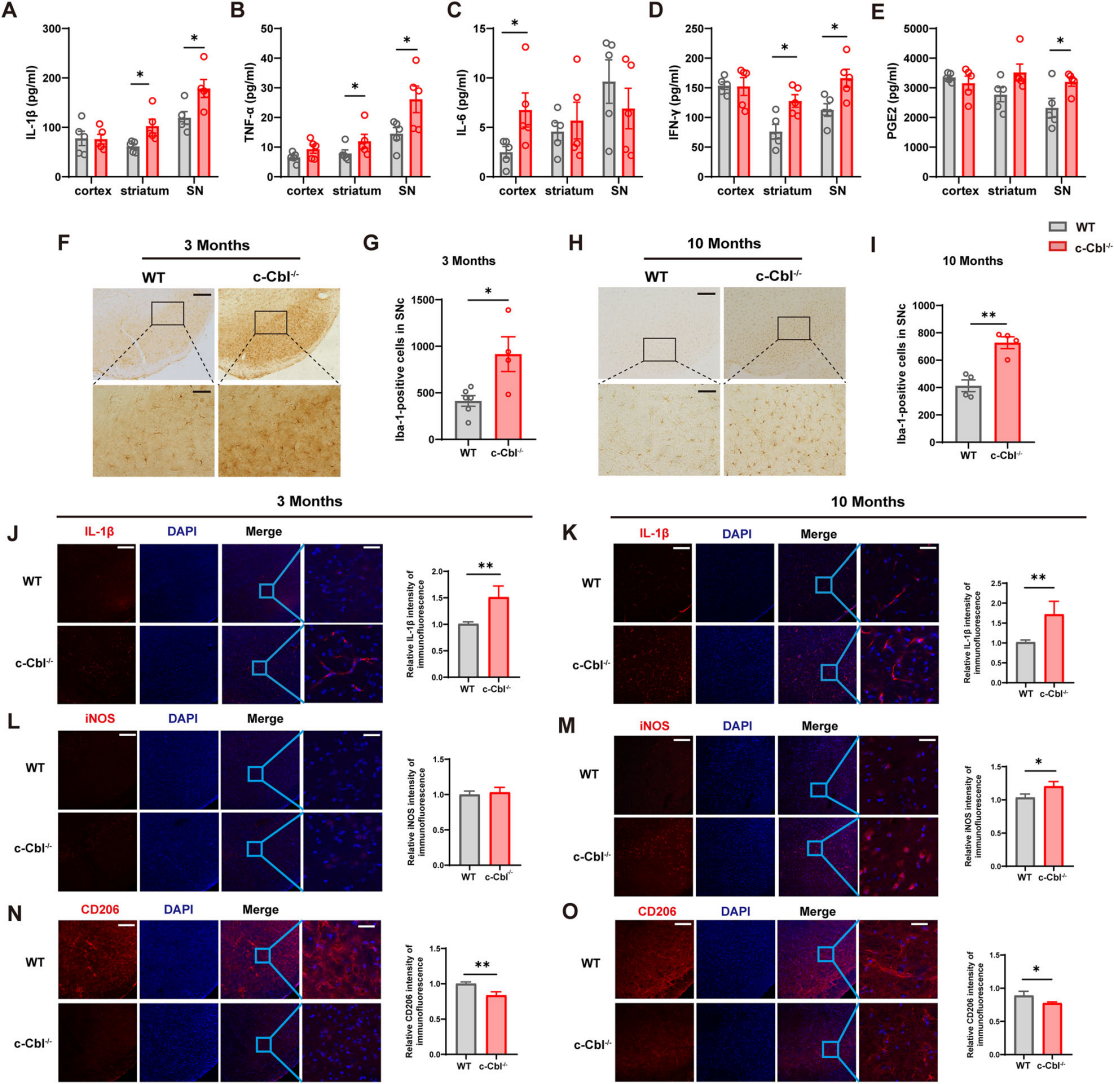
c-Cbl deficiency enhances the levels of proinflammatory cytokines and microglial activation in the substantia nigra. **A**–**E** Proinflammatory cytokine levels in cortical, striatal, and nigral tissues measured by ELISA: (**A**) IL-1β, (**B**) TNF-α, (**C**) IL-6, (**D**) IFN-γ, (**E**) PGE2 (*n* = 5). **F**–**I** Microphotographs and quantification of Iba-1-positive microglia in the SNc of 3 month-old and 10 month-old c-Cbl^-/-^ mice and WT mice (*n* = 4-6). Scale bars: 200 μm (above) or 50 μm (below). **J**–**O** Representative immunofluorescence staining of IL-1β (**J**–**K**), iNOS (**L**–**M**), and CD206 (**N**–**O**) in the SNc of 3 month-old and 10 month-old c-Cbl^-/-^ mice and WT mice (*n* = 4), and the intensity was analyzed by ImageJ. Scale bars: 100 μm (left) or 25 μm (right). Data expressed as mean ± SEM; *p < 0.05, **p < 0.01 vs. age-matched WT.

We also measured dopamine (DA) and its metabolites, dihydroxyphenylacetic acid (DOPAC) and homovanillic acid (HVA), in the striatum via HPLC. In 3 month-old c-Cbl^-/-^ mice, DA and HVA levels were significantly decreased, while in 10 month-old c-Cbl^-/-^ mice, only HVA levels were significantly reduced (Fig. 2F–H). The DA metabolism rate, represented by the (DOPAC + HVA)/DA ratio, was increased in the striatum of 3 month-old c-Cbl^-/-^ mice (Fig. 2I). However, no significant change was observed in 10 month-old mice, possibly due to decreased DA levels in the WT group. Immunohistochemical analysis of TH in the SNc indicated a reduction in TH-positive cells in both 3 month-old and 10 month-old c-Cbl^-/-^ mice compared to those in WT mice (Fig. 3J–K). Additionally, the number of TH-positive fibers in the striatum was significantly reduced in 10 month-old WT mice compared to 3 month-old WT mice (Fig. S1). These results demonstrate that c-Cbl deficiency leads to motor deficits, decreased striatal dopamine, and loss of dopaminergic neurons in the SN.

### c-Cbl deficiency enhances the levels of proinflammatory cytokines and microglial activation in the substantia nigra

As we reported that c-Cbl was more highly expressed in primary microglia than in neurons, indicating its role in brain innate immunity, we examined the levels of proinflammatory cytokines and chemokines (IL-1β, IL-6, TNF-α, IFN-γ, and PGE2) in the cortex, striatum, and SN via ELISA. c-Cbl^-/-^ mice showed significantly elevated levels of IL-1β, TNF-α, IFN-γ, and PGE2 in the SN and elevated levels of IL-1β, TNF-α, and PGE2 in the striatum compared to those in WT mice (Fig. 3A–E). Given that microglia-mediated neuroinflammation plays crucial roles in DA neuron death, we investigated microglial activation by using immunohistochemistry to detect the marker Iba-1 and assess morphological microglial activation in the SNc. A large number of activated microglia with enlarged cell bodies were observed in the SNc of c-Cbl knockout mice compared to those in the SNc of WT mice in both age groups (Fig. 3F–I).

Microglial phenotypes can be distinguished by the expression of characteristic surface marker genes [22]. Immunofluorescence analysis revealed significant increases in the M1 markers IL-1β and iNOS in the SNc of 10 month-old c-Cbl^-/-^ mice and an increase in IL-1β in 3 month-old c-Cbl^-/-^ mice (Fig. 3J–M). Conversely, the fluorescence intensity of the M2 marker CD206 was significantly reduced in both age groups (Fig. 3N, O). These findings suggest that c-Cbl deletion promotes proinflammatory cytokine release and microglial activation in the SN, facilitating a shift from the M2 to M1 microglial phenotype.

### Knockdown of c-Cbl expression in the substantia nigra induces motor deficits and exacerbates the death of dopaminergic neurons

**Fig. 4:**
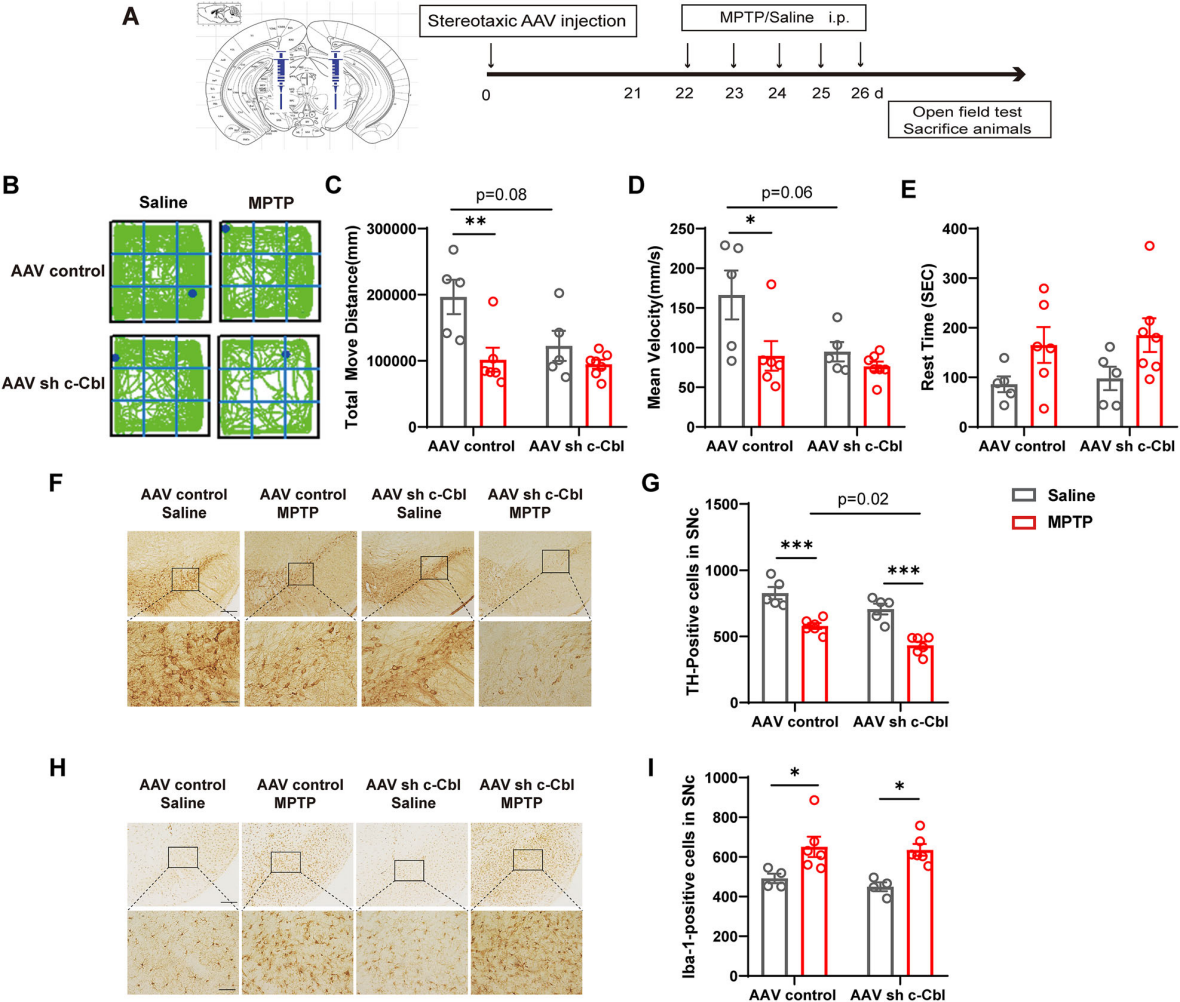
Knockdown of c-Cbl expression in the substantia nigra induces motor deficits and exacerbates the death of dopaminergic neurons. **A** Diagram of the experimental design. Three weeks after stereotactic injection of adeno-associated virus (AAV) c-Cbl shRNA into the SNc, the mice were intraperitoneally injected with a single dose of MPTP (20 mg/kg) or saline daily for 5 consecutive days. Motor behaviors were analyzed after MPTP injection. **B** Representative movement trajectories in the open field test. **C**–**E** Changes in locomotor behavior in AAV control and AAV sh c-Cbl mice treated with saline or MPTP (*n* = 5-7): (**C**) total movement distance, (**D**) mean velocity and (**E**) rest time were recorded in 30 min. **F** Microphotographs of TH-positive neurons in the SNc of AAV control and AAV sh c-Cbl mice treated with saline or MPTP. **G** Quantification of TH-positive neurons in the SNc (*n* = 5-6). **H** Microphotographs of Iba-1-positive cells in the SNc of each group. **I** Quantification of Iba-1-positive neurons in the SNc of each group (*n* = 4-6). Scale bars: 200 μm (above) or 50 μm (below). Data expressed as mean ± SEM; *p < 0.05, **p < 0.01, ***p < 0.001 vs. respective control.

To further examine the effects of c-Cbl deletion on Parkinsonism, we knocked down c-Cbl expression in the SNc via stereotactic injection of an adeno-associated virus (AAV). Three weeks post-injection, the mice received MPTP (20 mg/kg) or saline intraperitoneally for five consecutive days (Fig. 4A). Fluorescence colocalization of ZS green with TH and Iba-1 in the SNc showed that the virus infected both DA neurons and microglia (Fig. S2A, B). In the open field test, MPTP injection significantly reduced the total distance traveled and average speed of the mice. In the saline-treated groups, the mice in the AAV sh c-Cbl group exhibited decreased movement compared to those in the AAV control group (Fig. 4B–E).

TH-positive cell counts in the SNc indicated that MPTP significantly decreased these cells, with increased susceptibility to MPTP-induced neurotoxicity in the AAV sh c-Cbl group (Fig. 4F, G). Additionally, the number of TH-positive fibers in the striatum was significantly reduced only in the AAV control group after MPTP treatment, but not in the AAV sh c-Cbl group (Fig. S2C, D). Iba-1 immunohistochemistry revealed extensive microglial overactivation in both genotypes post-MPTP injection, with no exacerbation following c-Cbl knockdown (Fig. 4H, I). These results suggest that c-Cbl knockdown in the SN aggravates motor impairment and accelerates DA neuron degeneration in the MPTP mouse model.

### Transcriptomic profiling of the substantia nigra reveals c-Cbl-dependent microglial regulation

**Fig. 5:**
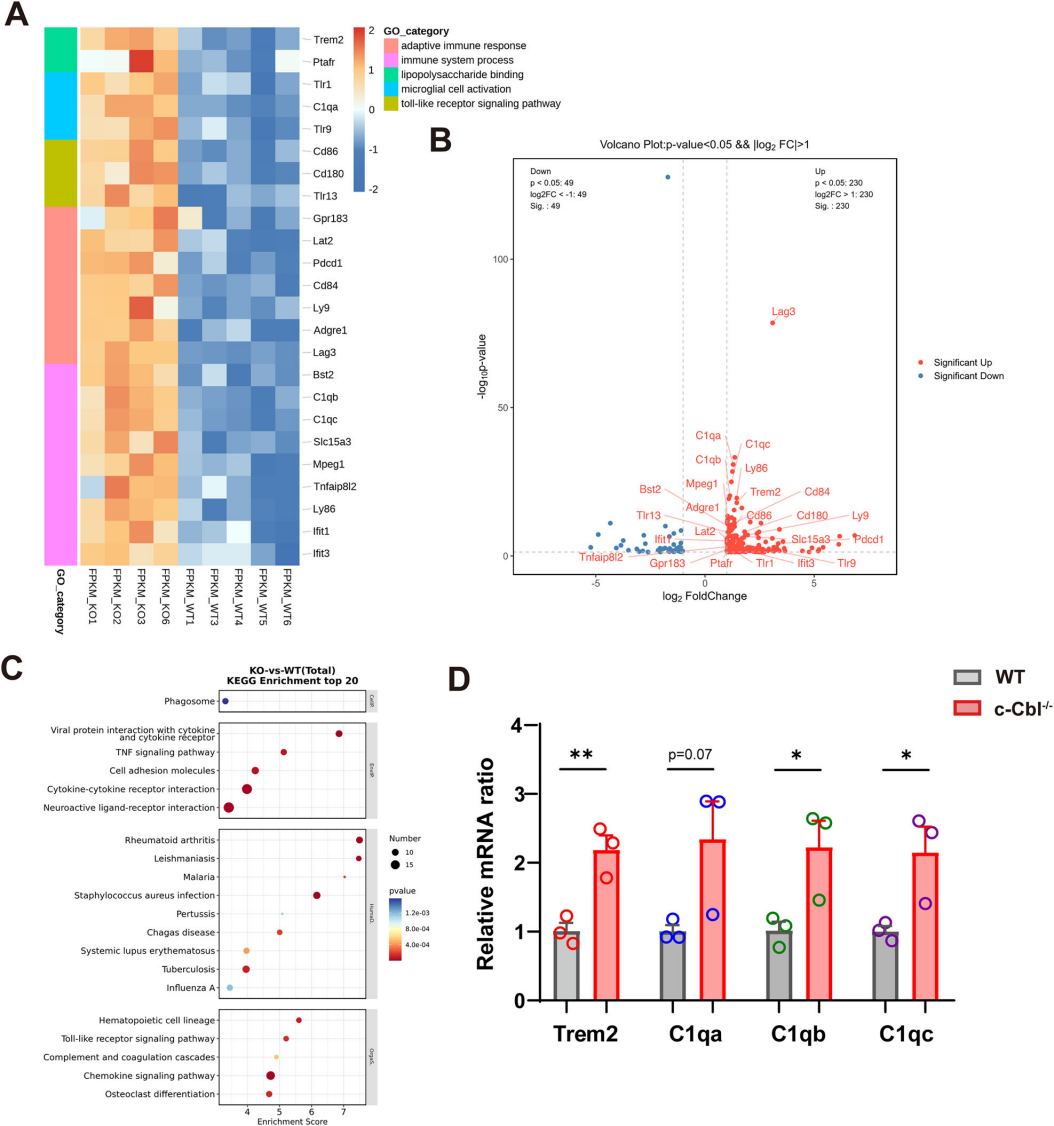
Transcriptomic profiling of the substantia nigra reveals c-Cbl-dependent microglial regulation. **A** Heatmap and GO analysis of microglia-related differentially expressed genes (DEGs) between 3 month-old WT and c-Cbl^-/-^ mice (*n* = 4-5). **B** Volcano plot of DEGs in the SN of WT and c-Cbl^-/-^ mice. **C** The top 20 enriched KEGG pathways are displayed as bubble charts. **D** qPCR validation of Trem2, C1qa, C1qb and C1qc expression in the SN of WT and c-Cbl^-/-^ mice (*n* = 3). Data expressed as mean ± SEM; *p < 0.05, **p < 0.01 vs. WT group.

To elucidate the molecular mechanisms of c-Cbl, RNA sequencing (RNA-seq) analysis was performed on the SN of WT and c-Cbl^-/-^ mice. DEGs with *p* < 0.05 and a |log2 ratio | ≥ 1 were identified, revealing 230 upregulated and 49 downregulated genes in c-Cbl^-/-^ mice (Fig. S3A; Supplementary material 2). Principal component analysis and gene ontology (GO) analysis highlighted significant changes in genes related to microglial activation, immune response, and inflammatory signaling (Fig. 5A, B; Fig. S3B). KEGG pathway enrichment analyses revealed significant enrichment in pathways such as chemokine signaling, toll-like receptor signaling, and TNF signaling (Fig. 5C; Fig. S3C). The RNA-seq results were confirmed by qPCR with selected upregulated genes in the SN (Fig. 5D). Triggering receptor expressed on myeloid cells 2 (TREM2) is predominantly and exclusively expressed by microglia in the brain, and it has been identified as a novel risk gene for sporadic PD [23]. Microglia are pivotal in central nervous system disorders involving complement component 1q (C1q). Upon stimulation by exogenous C1q, microglia produce endogenous C1q, thereby activating additional microglia. This process creates a positive feedback loop that exacerbates inflammation [24, 25]. Notably, both TREM2 and C1q, which are critical microglial genes, were found to be significantly upregulated in c-Cbl knockout mice. This upregulation suggests a significant role for c-Cbl in the regulation of microglial activation and inflammatory responses.

### c-Cbl deletion facilitates the switch of microglial phenotypes from M2 to M1 via PI3K/Akt signaling pathway

**Fig. 6:**
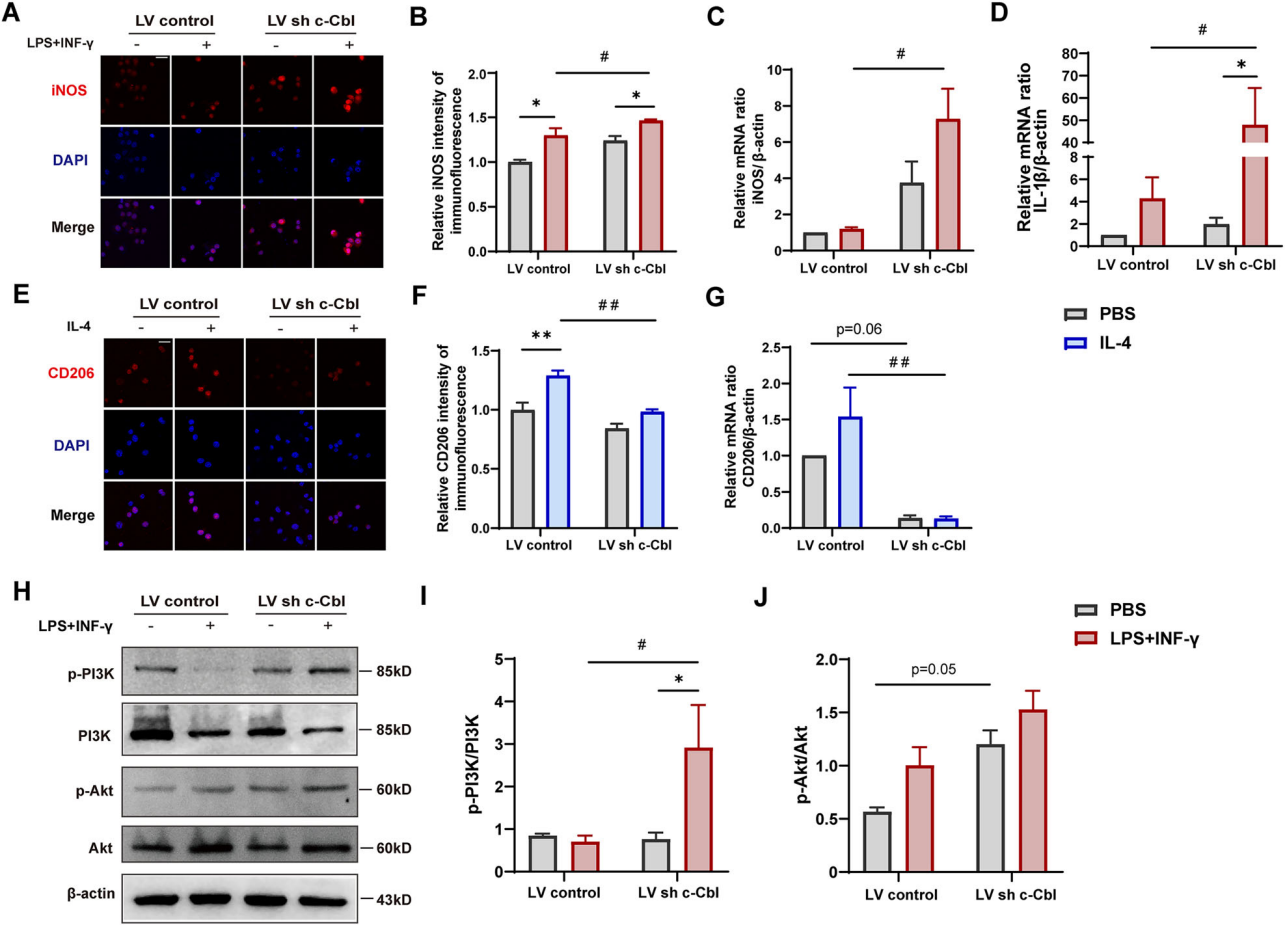
c-Cbl deletion facilitates the switch of microglial phenotypes from M2 to M1 via PI3K/Akt signaling pathway. **A** iNOS immunofluorescence in BV2 microglia with c-Cbl knocked down by lentiviral vectors treated with or without LPS + INF-γ. **B** Quantitative of iNOS fluorescence intensity using ImageJ. **C** qPCR analysis of the M1 marker iNOS in each group. **D** qPCR analysis of the M1 marker IL-1β in each group. **E** CD206 immunofluorescence in BV2 microglia with c-Cbl knocked down by lentiviral vectors treated with or without IL-4. **F** Quantitative of CD206 fluorescence intensity using ImageJ. **G** qPCR analysis of the M2 marker CD206 in each group. (**H**–**J**) Representative immunoblot and quantitative analysis of the phosphorylation of PI3K and Akt and total PI3K and total Akt. Scale bars: 100 μm. Data expressed as mean ± SEM of four independent experiments; *p < 0.05, **p < 0.01 versus PBS group; ^#^*p* < 0.05, ^##^*p* < 0.01, versus negative control group.

To explore the role of c-Cbl in microglial polarization, BV2 microglia were transfected with lentiviral vectors carrying c-Cbl shRNA (LV sh c-Cbl) and stimulated with LPS + IFN-γ or IL-4. Immunofluorescence analyses revealed increased expression of the M1 marker iNOS in knockdown cells after LPS + IFN-γ treatment (Fig. 6A, B). Consistently, the LPS + INF-γ-induced increase in the mRNA expression of M1 markers, including iNOS (Fig. 6C) and IL-1β (Fig. 6D), was significantly elevated in the c-Cbl-knockdown cells. Conversely, the fluorescence intensity of CD206 was significantly decreased post-IL-4 challenge (Fig. 6E, F). Consistent with the real-time PCR results, the expression of CD206 induced by IL-4 decreased upon c-Cbl knockdown (Fig. 6G). These findings indicate that c-Cbl knockdown promotes a shift from the M2 phenotype to the M1 phenotype.

Recent research has demonstrated that repressing c-Cbl expression leads to aberrant activation of the PI3K/Akt signaling pathway during the osteogenic differentiation of human adipose-derived mesenchymal stem cells [26]. Previous studies have shown that activation of PI3K and Akt plays an essential role in LPS-induced microglial activation [27]. To investigate whether c-Cbl knockdown affects PI3K/Akt activation during the switch of microglial phenotypes, we performed a series of experiments. As shown in Fig. 6H–J, phosphorylation of PI3K was significantly greater in the c-Cbl-knockdown microglia than in the negative control microglia after treatment with LPS + IFN-γ. Additionally, the phosphorylation of Akt was increased in c-Cbl-knockdown microglia treated with PBS. These results suggest that c-Cbl deletion facilitates the switch of microglial phenotypes from M2 to M1 through the PI3K/Akt signaling pathway.

### c-Cbl-deficient microglia promote neuron degeneration

**Fig. 7:**
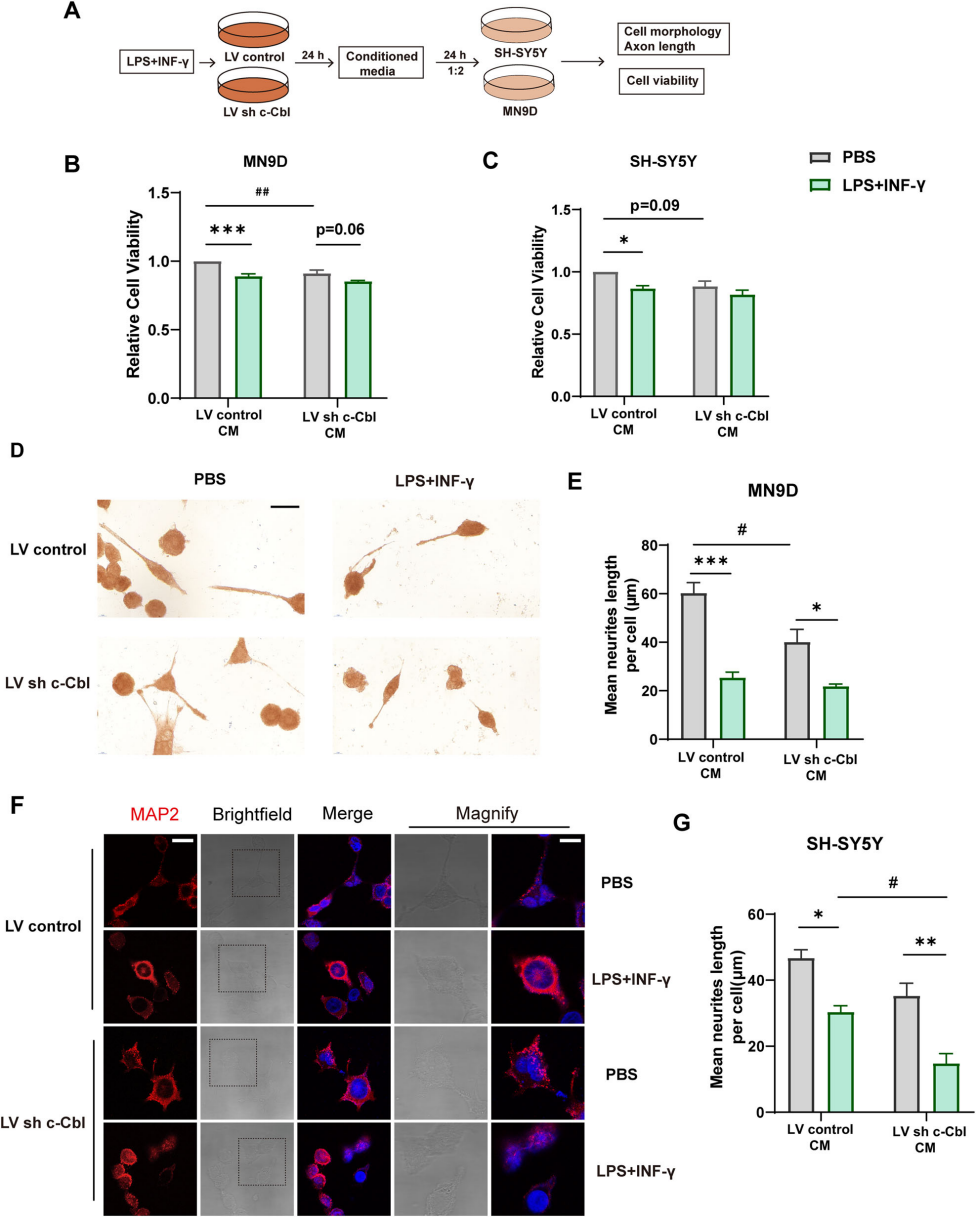
c-Cbl-deficient microglia promote neuron degeneration. **A** Experimental workflow for microglia-neuron coculture assays. **B** Cell viability of MN9D cells after treatment with conditioned medium (CM) from BV2 microglia with or without LPS + INF-γ stimulation. **C** Cell viability of SH-SY5Y cells after treatment with CM from BV2 microglia with or without LPS + INF-γ stimulation. **D**-**E** TH staining and measurement of neurite growth length in MN9D cells treated with CM from BV2 microglia with or without LPS + INF-γ stimulation. **F**-**G** MAP2 staining and measurement of neurite growth length in SH-SY5Y cells treated with CM from BV2 microglia with or without LPS + INF-γ stimulation. Scale bars: 100 μm (left) or 25 μm (right). Data expressed as mean ± SEM from four independent experiments; *p < 0.05, **p < 0.01, ***p < 0.001 vs. control; ^#^*p* < 0.05, ^##^*p* < 0.01 vs. negative control group.

Given the association between microglia-induced neuroinflammation and neuronal survival, we examined the impact of c-Cbl knockdown in microglia on neuronal damage (Fig. 7A). Mouse dopaminergic MN9D cells and human neuroblastoma SH-SY5Y cells were treated with LPS + IFN-γ-primed conditioned medium (CM) from BV2 microglia. CCK-8 assays showed decreased cell viability in both cell lines treated with LPS + IFN-γ-primed CM, with further reductions observed under c-Cbl knockdown conditions (Fig. 7B, C). Interestingly, even without LPS + INF-γ stimulation, CM from microglia with c-Cbl knockdown decreased cell viability in both cell lines.

TH immunohistochemical staining of MN9D cells and MAP2 immunofluorescence in SH-SY5Y cells were used to assess the survival of dopaminergic neurons. As shown in Fig. 7D–E, compared with those in the respective PBS-treated groups, the neurite length of the MN9D cells in the LPS + INF-γ-primed microglial CM group was significantly shorter. Consistently, LPS + INF-γ-primed microglial CM also shortened the neurite length of SH-SY5Y cells (Fig. 7F, G). These findings indicate that c-Cbl knockout induces reactive microglia-mediated neuronal damage and exacerbates dopaminergic neuron degeneration. Moreover, exposure to CM from LPS + INF-γ-primed microglia with c-Cbl knockdown further exacerbated neuronal damage. Because of the significant differences between the LV control and LV sh c-Cbl groups at baseline, the rate of change from the LPS + INF-γ group to the PBS group in the LV control group was greater than that in the LV sh c-Cbl group. Collectively, these results demonstrate that c-Cbl deficiency in microglia enhances inflammatory cytokine production and impairs neuronal survival under both baseline and LPS + IFN-γ-induced conditions.

## Discussion

Previous research has demonstrated that c-Cbl is involved in microglia-mediated neuroinflammation [17]. In this study, we observed that c-Cbl knockout leads to motor deficits, decreased dopamine levels in the striatum, and the loss of DA neurons in the SN of mice. c-Cbl deletion significantly promoted the release of proinflammatory cytokines and microglial activation in the SN, causing a switch from the M2 phenotype to the M1 phenotype. Additionally, c-Cbl knockdown in the SN exacerbates behavioral impairment and accelerates DA neuron degeneration in the MPTP mouse model. Finally, we demonstrated that c-Cbl deletion facilitates the switch of microglial phenotypes from M2 to M1 via the PI3K/Akt signaling pathway, impairing dopaminergic neuronal survival.

Our prior studies have shown that c-Cbl proteins are highly expressed in the normal SN and striatum. In this study, we found that c-Cbl levels decreased in the SN of aged mice and PD patients, suggesting a correlation between c-Cbl and PD pathology. Both young (3 month-old) and older (10 month-old) c-Cbl knockout mice exhibited behavioral deficits and pathological changes characteristic of PD, including decreased dopamine levels in the striatum and loss of DA neurons in the SN. These findings indicate that c-Cbl is closely related to the pathological process of PD. However, the specific functions of c-Cbl and the related molecular mechanisms involved in disease progression remain unclear.

Cbl proteins are E3 ubiquitin ligases implicated in regulating the functional activity of various immune cells [28]. Previous studies have shown that c-Cbl downregulates NF-κB signaling by catalyzing TRAF6 ubiquitination [20]. c-Cbl mediates the ubiquitination and degradation of RelB, playing a critical role in fungus-induced intestinal inflammation [21]. Phosphorylation of Cbl at Tyr371 suppresses NLRP3 inflammasome activation [18]. These findings indicate that c-Cbl plays a significant role in inflammation. As microglia are the primary resident immune cells in the CNS, microglial polarization plays a pivotal role in regulating inflammatory reactions. Microglial activation is often characterized by two polarization states: the classically activated M1 phenotype and the alternatively activated M2 phenotype. M1 microglia can be induced by substances such as IFN-γ and LPS, leading to the overexpression of inflammatory factors, reactive oxygen species (ROS), and nitric oxide (NO), which contribute to neurotoxicity. In contrast, M2 microglia, induced by anti-inflammatory factors such as IL-4, release anti-inflammatory cytokines, growth factors, and neurotrophic factors, providing anti-inflammatory and neuroprotective effects [29]. In our study, c-Cbl knockout significantly promoted the release of proinflammatory cytokines and microglial activation in the SN, increasing the expression of M1 markers (IL-1β and iNOS) and decreasing the expression of an M2 marker (CD206). These results suggest that c-Cbl regulates the switch of microglial phenotypes from M2 to M1.

Given that c-Cbl is essential for microglial activation, we further explored its role in PD pathogenesis. MPTP, an environmental toxin that induces parkinsonism, was used to establish a PD animal model [30]. AAV sh c-Cbl was injected into the SNc of mice to knock down c-Cbl expression, creating a subacute neurotoxin MPTP-induced PD model. Our results showed that the AAV sh c-Cbl group exhibited greater DA neuron loss in the SNc and motor deficits even without MPTP treatment than did the AAV control group. However, the number of Iba-1 positive microglia did not increase following c-Cbl knockdown. This observation suggests that the change in microglial activity is not due to proliferation but rather a marked change in morphology. Specifically, microglia adopt an ameboid shape characterized by a larger and more rounded cell body with fewer and shorter processes. This morphological transformation is indicative of a phagocytic state and is associated with proinflammatory functions. Our findings clearly demonstrated that c-Cbl deficiency in the SN exacerbates DA neuron death in the MPTP-induced PD model.

RNA sequencing analysis of total RNA extracted from the SNc of c-Cbl knockout and WT mice revealed significant increases in the expression of innate immunity signaling genes and genes related to microglial phenotype and function, such as Aif1, C1q, Ctss, Lag3, Ly86, Oasl2, Ptpn6, Trem2, and Tyrobp [31–37]. TREM2, an immune receptor expressed by microglia in the brain, plays an essential role in neurodegenerative diseases such as Alzheimer’s disease (AD) and PD [38]. TREM2 expression was significantly increased in the SN of c-Cbl knockout mice exhibiting PD-like symptoms and microglial activation. Previous studies have shown that TREM2 expression increases in inflammatory states in vivo but decreases in vitro upon inflammatory stimulation [39]. However, the relationship between TREM2 and c-Cbl remains uncertain. Our RNA-seq data suggest that c-Cbl knockout activates the TLR signaling pathway, implicating several inflammatory cytokines in this mechanism.

Another important finding is that c-Cbl plays a crucial role in PD pathogenesis. Microglia-mediated neuroinflammation inversely correlates with DA neuron survival in PD patients [40]. Activated microglia surrounding DA neurons often exhibit M1 phenotypes, releasing cytokines such as IL-1β, iNOS, TNF-α, and IL-6 [41]. To determine whether this proinflammatory role of microglia affects surrounding neurons, we treated mouse dopaminergic MN9D cells and human neuroblastoma SH-SY5Y cells with LPS + INF-γ-primed conditioned medium from BV2 microglia. The results showed decreased cell viability and shortened neurite length in both cell lines, especially with CM from c-Cbl-knockdown cells. Our results demonstrated that c-Cbl deficiency in microglia enhances LPS + INF-γ-induced inflammatory cytokine production, subsequently impairing dopaminergic neuronal survival.

The most significant finding of this study is that c-Cbl knockdown induces a switch in microglia from the beneficial M2 phenotype to the detrimental M1 phenotype via the PI3K/Akt signaling pathway, ultimately accelerating the death of DA neurons during PD progression (Fig. S4).

In conclusion, our findings demonstrated that c-Cbl knockout induces PD-like symptoms and microglial activation. c-Cbl regulates the switch of microglia from the detrimental M1 phenotype to the beneficial M2 phenotype, potentially contributing to the immune pathogenesis of PD. Therefore, c-Cbl may be a promising therapeutic target for PD.

## Materials and methods

### Animals

c-Cbl knockout (c-Cbl^-/-^) mice were generated as previously described [42]. Genotyping was conducted via polymerase chain reaction (PCR) analysis of DNA extracted from tail biopsies. Both c-Cbl^-/-^ mice and their wild-type (WT) littermate controls were bred and maintained in a specific pathogen-free (SPF) facility at Capital Medical University. All the animals were maintained on a 12 h light/dark cycle at 20°C to 23°C with free access to food and water. All animal experimental procedures were approved by the Institutional Animal Care and Use Committee (IACUC) of the Capital Medical University (AEEI-2021-097) and were performed in strict accordance with the “Regulations for the Administration of Affairs Concerning Experimental Animals (State Science and Technology Commission, China, 1988). Sample sizes were determined using G*Power software. For behavioral testing and monoamine neurotransmitters, a one-way ANOVA with effect size *f* = 0.4 (estimated from literature), *α* = 0.05, and power = 0.95 required 10 mice per group. No animals were excluded from the analysis in this study.

### Subacute MPTP mouse model

C57BL/6 J male mice were randomly assigned to two groups: the control group and the MPTP model group. To construct the PD model, the mice in the MPTP group received intraperitoneal (i.p.) injections of MPTP (25 mg/kg, dissolved in 0.9% saline) once daily for five consecutive days, while the control group was given an equal volume of normal saline (i.p.). The control group was administered an equivalent volume of 0.9% saline (i.p.) under the same schedule. Two days following the final MPTP injection, behavioral assessments were conducted to evaluate the impact of MPTP treatment. Subsequently, all animals were humanely euthanized, and tissues were harvested for further biochemical and histological analyses.

### Open field test

To investigate the effect of c-Cbl knockout on the locomotor abilities of mice, an open field test was conducted. Mice were placed in a square box with an enclosure, and their movement trajectories were recorded. The movement parameters, including total distance traveled, average speed, and resting time during a 30 min period of free movement, were analyzed using SuperMaze V2.0 software (Xin Ruan, Shanghai, China).

### Rotarod test

The rotarod test was used to assess motor coordination in PD models. Mice were placed on an accelerating rod that increased from 4 to 50 rpm over a 5 min period. Prior to the test, the mice underwent a 3 day training regimen. During the official test, each mouse was tested three times with intervals of at least 30 min to ensure adequate rest. The average performance from the three trials was used for analysis.

### High-performance liquid chromatography (HPLC)

DA and its metabolites, DOPAC and HVA, were quantified in the striatum using HPLC with an electrochemical detector (Model 5600 A CoulArray Detector System, ESA, MA, USA). Tissue samples were homogenized in 200 mM ice-cold perchloric acid and incubated in an ice bath for 60 min. Following centrifugation at 13,000 × g for 20 min at 4°C, the supernatant was transferred to a clean tube and mixed with a solution containing 20 mM potassium citrate, 300 mM potassium dihydrogen phosphate, and 2 mM EDTA-Na2 to precipitate the perchloric acid. After another 60 min incubation in an ice bath and a second centrifugation, the supernatant was filtered through a 0.22 μm filter and injected into the HPLC system. The mobile phase consisted of 125 mM sodium citrate buffer with 20% methanol, 0.1 mM EDTA-Na2, and 0.5 mM 1-octanesulfonic acid sodium salt, with a flow rate of 1.2 mL/min.

### Immunohistochemistry

Brain samples were collected and postfixed in 4% paraformaldehyde (PFA) at 4°C overnight, followed by immersion in 20% sucrose in phosphate-buffered saline (PBS) until the brains sank and then in 30% sucrose until they sank again. Coronal sections (40 μm) were cut using a freezing microtome (Leica, Germany) and stored in an antifreeze solution. Tyrosine hydroxylase (TH) and ionized calcium-binding adapter molecule 1 (Iba-1) were detected using a mouse monoclonal antibody against TH (T1299, Sigma, USA) and a rabbit antibody against Iba-1 (019-19741, FUJIFILM Wako, USA). Immunoreactivity was visualized with DAB substrate-chromogen solution (ZSGB-BIO, Beijing, China). The number of TH- and Iba-1-immunoreactive cells in the SNc of the midbrain was quantified using ImageJ.

### Immunofluorescence

For immunofluorescence staining, the sections were blocked with 5% goat serum (ZSGB-BIO, Beijing, China) for 1 h at room temperature and then incubated overnight at 4°C with the following primary antibodies: anti-TH (T1299, Sigma), anti-IL-1β (201-LB-005/CF, R&D Systems), anti-iNOS (18985-1-AP, Proteintech), and anti-CD206 (18704-1-AP, Proteintech). The sections were then incubated with Alexa Fluor 594-conjugated anti-mouse IgG secondary antibody (8890S, Cell Signaling Technology) or Alexa Fluor 594-conjugated anti-rabbit IgG secondary antibody (8889S, Cell Signaling Technology) for 1 h at room temperature. After washing, the sections were counterstained with DAPI and imaged using a Leica SP8 confocal microscope. Images were processed and analyzed using ImageJ.

### Western blot

Brain tissue was homogenized in RIPA lysis buffer supplemented with 1% protease inhibitor and incubated on ice for 30 min. The supernatant was collected after centrifugation at 13,000 rpm for 15 min at 4°C. The protein concentration was determined using a BCA protein assay kit (23227, Pierce, Thermo Fisher Scientific, Inc.). Proteins were denatured at 95°C for 5 min, separated by 10% SDS‒PAGE, and transferred to membranes. The membranes were blocked with 5% skim milk for 1 h and incubated with primary antibodies overnight at 4°C and then with HRP-conjugated secondary antibodies. Bands were visualized using a chemiluminescent substrate and analyzed with ImageJ.

### Enzyme-linked immunosorbent assay (ELISA)

The cortex, striatum, and midbrain were homogenized in ice-cold lysis buffer (20 mM Tris, 0.25 M sucrose, 2 mM EDTA, 10 mM EGTA, 1% Triton X-100, and a protease inhibitor cocktail). The samples were centrifuged at 100,000 × g for 40 min. The levels of IL-1β, IL-6, TNF-α, interferon-γ (IFN-γ), and prostaglandin E2 (PGE2) were measured using ELISA kits (ExCell Bio, Shanghai, China) following the manufacturer’s protocols.

### RNA sequencing

Total mRNA was extracted from the SN tissues of WT and c-Cbl^-/-^ mice. RNA libraries were sequenced on the Illumina platform by OE Biotech Co., Ltd. (Shanghai, China). Differentially expressed genes were defined as those with fold changes of |log2 ratio | ≥ 1 and *p* < 0.05. Gene Ontology (GO) and Kyoto Encyclopedia of Genes and Genomes (KEGG) pathway enrichment analyses were conducted using the Metascape platform (https://Metascape.org/gp/index.html#/main/step1).

### Stereotaxic injection

Adeno-associated viral solutions were injected bilaterally into the SNc region using a 10 μL Hamilton syringe. A volume of 3 μL/side was injected at a rate of 0.1 μL/min. The stereotaxic coordinates (flat skull position) were AP = -3.2 mm, ML = ± 1.2 mm, and DV = -4.5 mm, based on the Paxinos and Franklin atlas (2001). AAV GFP and AAV sh c-Cbl viruses, purchased from Bio-lifespan (Bio-lifespan Co., LTD, Shanghai, China), had titers of 2.09 × 10¹³ and 1.35 × 10¹³, respectively.

### Cell culture and treatment

Mouse microglia (BV2, CRL-2647) and human neuroblastoma cells (SH-SY5Y, CRL-2266) were purchased from American Type Culture Collection (ATCC, USA). Mouse dopamine neuron cells (MN9D, CP-M207) were purchased from Wuhan Pricella Biotechnology Co., Ltd. BV2 cells were cultured in DMEM/F12 medium (Gibco, Thermo Fisher Scientific, USA) supplemented with 10% fetal bovine serum (FBS) and 1% penicillin-streptomycin (Gibco, Thermo Fisher Scientific, USA). SH-SY5Y and MN9D cells were cultured in their respective optimized media. For M1 phenotype induction, BV2 cells were treated with 100 ng/mL LPS (*Escherichia coli* 0111: B4, L4391, Sigma, USA) and 20 ng/mL IFN-γ (315-05, PeproTech, USA) for 24 h. For M2 phenotype induction, BV2 cells were treated with 20 ng/mL IL-4 (214-14, PeproTech, USA) for 24 h.

### Lentiviral vector transfection

Two oligonucleotide pairs targeting mouse c-Cbl were synthesized (Bio-lifespan Co., LTD, Shanghai, China) and ligated into lentiviral plasmids. Positive clones were selected via PCR. BV2 microglia at 70–80% confluence in 24-well plates were transfected with 3 μL of lentiviral vectors expressing green fluorescence and anti-puromycin proteins. Fluorescence intensity and anti-puromycin tests ensured virus titration and infection efficiency.

### Quantitative real-time PCR

Total RNA was extracted from BV2 cells using an RNA Easy Fast Tissue/Cell Kit (TIANGEN BioTech, Beijing, China). cDNA was synthesized from 1 μg of RNA using the FastKing RT Kit (TIANGEN BioTech, Beijing, China). qPCR was performed with PowerUp™ SYBR™ Green Master Mix (Applied Biosystems, Waltham, USA) in an ABI 7300 Fast Real-Time PCR System (Applied Biosystems, Waltham, USA). Gene expression was normalized to that of β-actin and calculated using the 2^−ΔΔCt^ method. The primers used were as follows: mouse-IL-1β- forward: 5’- TCATTGTGGCTGTGGAGAAG-3’; mouse-IL-1β- reverse: 5’- AGGCCACAGGTATTTTGTCG-3’; mouse-iNOS- forward: 5’- AATGGCAACATCAGGTCGGCCATCACT-3’; mouse-iNOS- reverse: 5’- GCTGTGTGTCACAGAAGTCTCGAACTC-3’; mouse-CD206- forward: 5’-GCAGGTGGTTTATGGGATGT-3’; mouse-CD206- reverse: 5’-GGGTTCAGGAGTTGTGG-3’; mouse-β-actin- forward: 5’- TGCTGTCCCTGTATGCCTCT-3’; and mouse-β-actin- reverse: 5’- TTGATGTCACGCACGATTTC-3’.

### Neurotoxicity assay

BV2 cells were plated in a 6-well plate at 1 × 10^6^ cells/well, and SH-SY5Y and MN9D cells were plated in 96-well plates at 5,000 cells/well or 24-well plates at 50,000 cells/well. BV2 cells were treated with 100 ng/mL LPS and 20 ng/mL IFN-γ for 24 h or with 20 ng/mL IL-4 for 24 h. The conditioned media (CM) from BV2 cells was used to treat SH-SY5Y and MN9D cells for 24 h. Neurotoxicity was quantified using a cell proliferation and cytotoxicity assay kit (CA1210, Solarbio Life Sciences, Beijing, China), and immunofluorescence data were normalized to those of LPS + IFN-γ-treated cells and CM from untreated BV2 cells.

### Statistical analysis

All experimental procedures were performed in triplicate to ensure reproducibility. To ensure unbiased evaluation, the experimenters responsible for treating the mice and collecting samples randomly assigned the samples to other experimenters for blinded analysis. The results were independently analyzed and validated by two additional experimenters. All the data are presented as the means ± standard error of mean (SEM). Statistical analyses were performed using Graph Pad Prism version 8.0 software (La Jolla, CA, USA). One-way or two-way analysis of variance (ANOVA) followed by multiple comparison post hoc tests or two-tailed unpaired Student’s *t*-tests were used as appropriate. Statistical significance was set at *P* < 0.05.

## Supplementary information

Supplementary figures

DEGs up- and downregulated in the substantia nigra of WT and c-Cbl knockout mice by RNA-seq analysis.

The full length uncropped original western blots used in this manuscript.

## Data Availability

The datasets generated or analyzed during this study are included in this published article or available from the corresponding author on reasonable request.
