# Supplementary material for: The E3 ligase c-Cbl modulates microglial phenotypes and contributes to Parkinson’s disease pathology
Source: Cell Death Discov. 2025 Apr 17;11:184. doi: 10.1038/s41420-025-02482-0 (PMC12006326; doi:10.1038/s41420-025-02482-0)
Supplement: Supplementary file 1 — Supplementary figures [file 41420_2025_2482_MOESM1_ESM.docx]

**Supplementary figures**

**
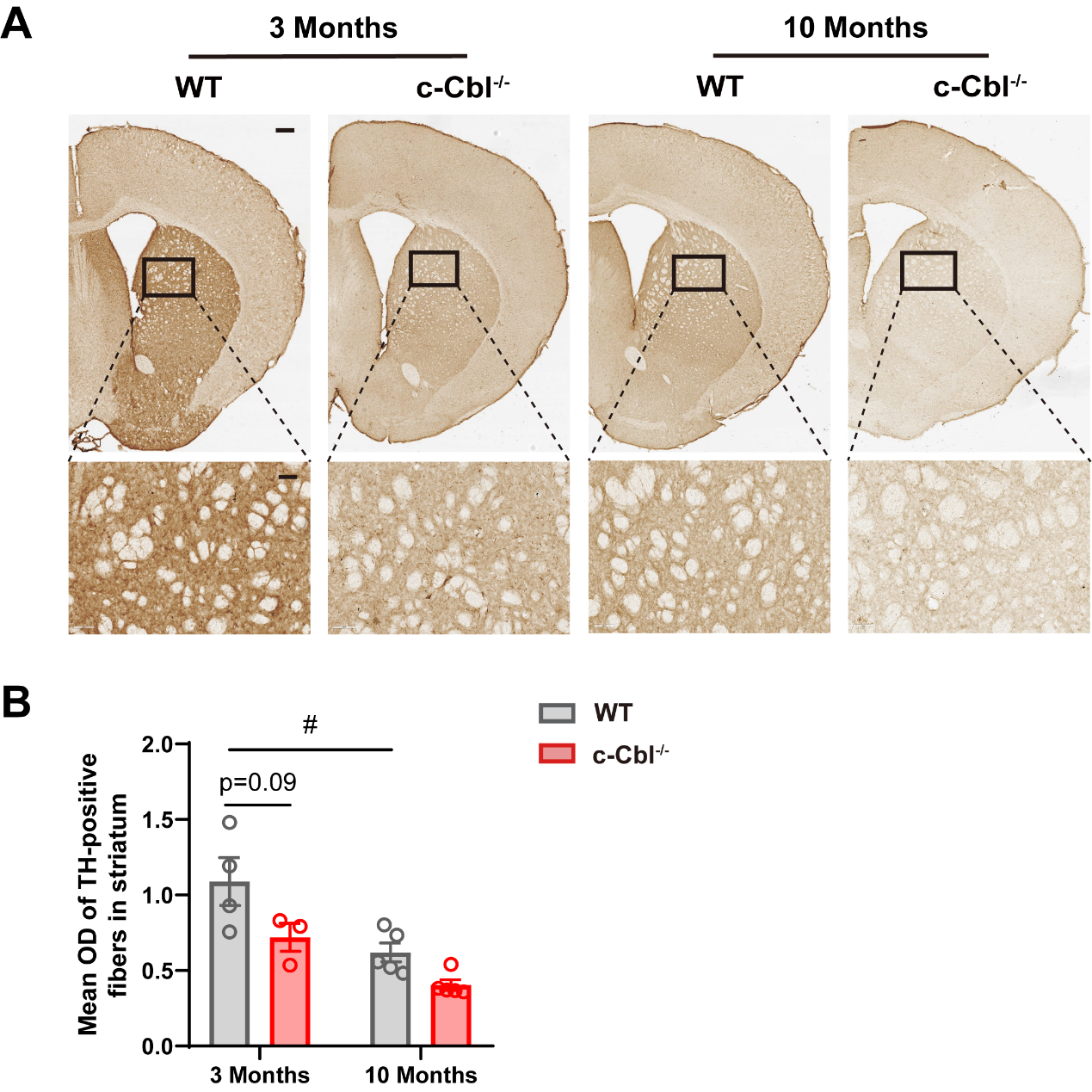
**

**Fig.S1.** **c-Cbl deficiency decreased dopamine** **fibers in the striatum.** (A) Microphotographs of TH-positive fibers in the striatum of 3-month-old and 10-month-old c-Cbl knockout mice and wild-type mice. (B) Mean density of striatal TH-positive fibers (n = 4-5). Scale bars: 500 μm (above) or 80 μm (below). Data expressed as mean ± SEM; ^#^p < 0.05 vs. 3-month-old WTmice.

**
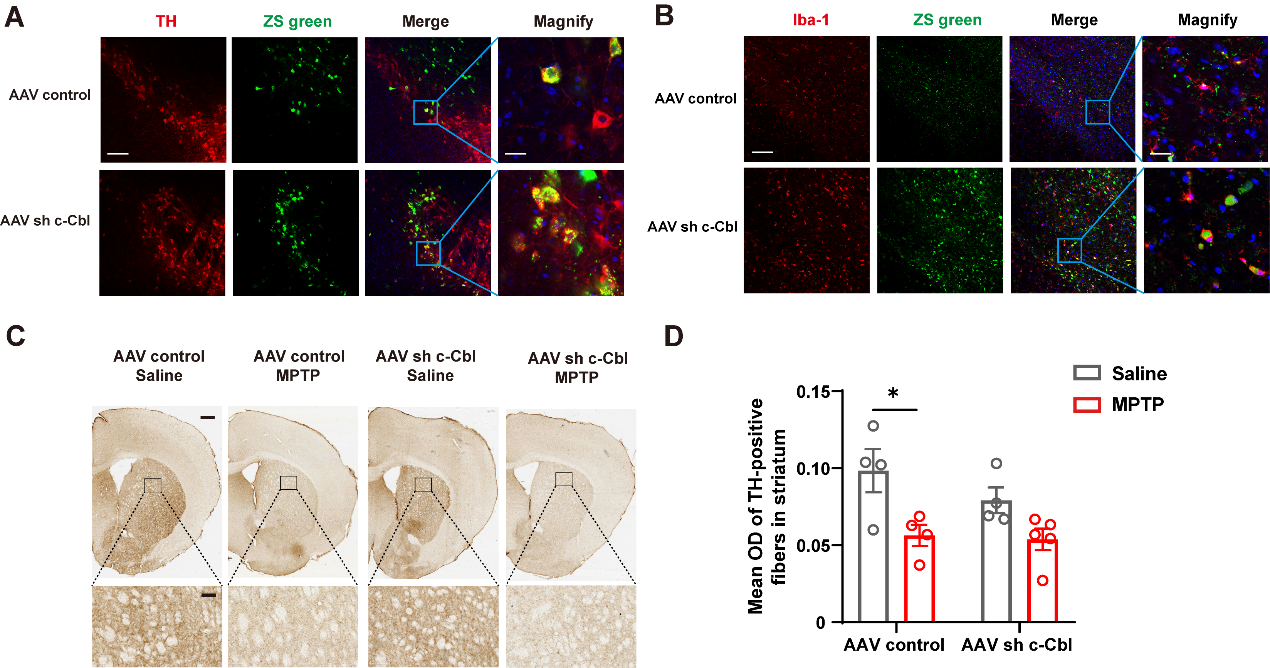
**

**Fig.S2. c-Cbl expression in the substantia nigra was knocked down by stereotactic injection of AAV-shRNA-ZS green.** (A) Colocalization of TH (red fluorescence) with ZS green in the SNc. Scale bars: 100 (left) or 50 μm (right). (B) Colocalization of Iba-1 (red fluorescence) with ZS green in the SNc. Scale bars: 100 (left) or 50 μm (right). (C) Microphotographs of TH-positive fibers in the striatum of AAV control and AAV sh-c-Cbl mice treated with saline or MPTP. (D) Mean density of striatal TH-positive fibers (n = 4-5). Scale bars: 500 μm (above) or 80 μm (below). Data expressed as mean ± SEM; *p < 0.05 vs. the respective control.

**
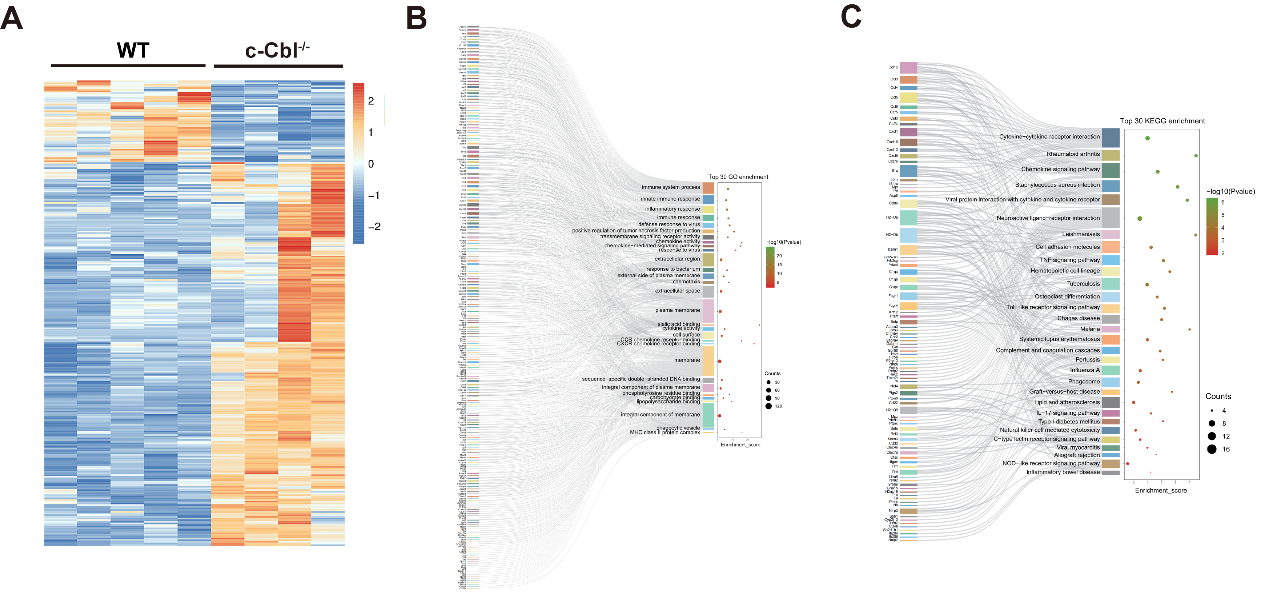
**

**Fig.S3. Transcriptome analysis of the substantia nigra in c-Cbl knockout mice compared to that in wild-type mice.** (A) Heatmap of differentially expressed genes in wild-type and c-Cbl knockout mice according to RNA-seq. (B) The top 30 enriched GO terms are displayed as a Sankey diagram and bubble chart. (C) The top 30 enriched KEGG pathways are displayed as a Sankey diagram and bubble chart.


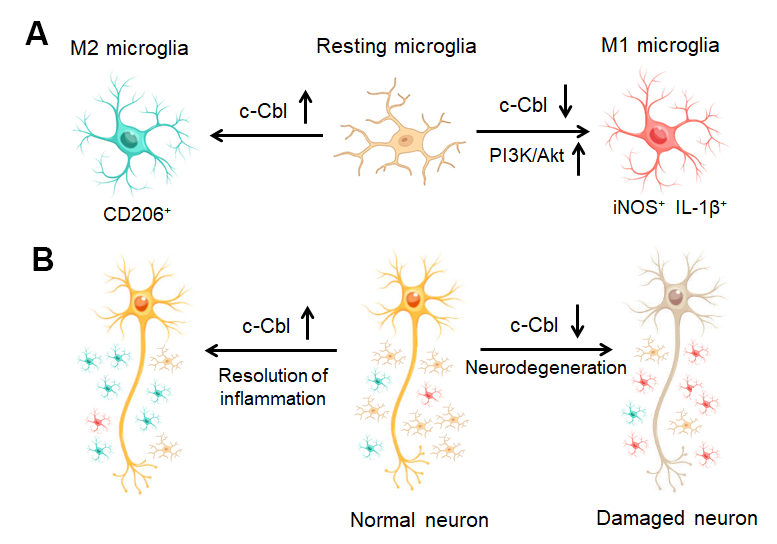


**Fig. S4. Proposed model of the involvement of c-Cbl in the regulation of microglial phenotypes and neurodegeneration.** (**A**) Under physiological conditions, microglia exist in a resting state characterized by a ramified morphology. Upon exposure to various immunological stimuli or injury, microglia can dramatically polarize into either the M1 or M2 phenotype. c-Cbl switches microglia from the detrimental M1 phenotype toward the beneficial M2 phenotype. Suppression of c-Cbl exacerbates M1 polarization through activation of the PI3K/Akt signaling pathway. (**B**) Suppression of c-Cbl impairs the neuroprotective effects of M2 microglia while concurrently amplifying the detrimental effects of M1 microglia. This shift results in an increase in the M1/M2 microglia ratio, which contributes to extensive neuronal death and exacerbates neurodegeneration.
