# Supplementary material for: The E3 ligase c-Cbl modulates microglial phenotypes and contributes to Parkinson’s disease pathology
Source: Cell Death Discov. 2025 Apr 17;11:184. doi: 10.1038/s41420-025-02482-0 (PMC12006326; doi:10.1038/s41420-025-02482-0)
Supplement: Supplementary file 2 — DEGs up- and downregulated in the substantia nigra of WT and c-Cbl knockout mice by RNA-seq analysis. [file 41420_2025_2482_MOESM2_ESM.pdf]

gene_id	BaseMean_DESeq2	BaseMean_control_WT	BaseMean_case_KO	FoldChange	log2FoldChange	p-value	q-value	Regulation
**Abhd12b**	154.5104855	60.21954526	272.3741608	4.523420917	2.177414249	0.002257237	0.058756903	Up
**Abi3**	115.231056	73.44354045	167.4654504	2.281131142	1.18974939	5.46E-11	2.06E-08	Up
**Acot5**	8.829933183	5.265701093	13.2852233	2.526118692	1.336922427	0.018986003	0.223147355	Up
**Acp5**	5.656570615	2.616327866	9.456874051	3.616387191	1.854549149	0.042906324	0.347954672	Up
**Adgre1**	124.6763759	85.54990445	173.5844652	2.027105858	1.01942143	4.25E-12	2.16E-09	Up
**Adgrg5**	14.13931877	6.2498919	24.00110236	3.839226935	1.94081584	0.001729598	0.047773312	Up
**Adora3**	9.917190988	5.060953428	15.98748794	3.163133251	1.661354332	0.001308688	0.039579253	Up
**Aif1**	53.90783759	32.97534271	80.0734562	2.429103741	1.280424105	1.83E-07	2.73E-05	Up
**Apol11b**	2.108089035	0.606009641	3.985688279	6.559478294	2.713581075	0.049841755	0.377778639	Up
**Arhgap45**	207.4430492	138.2310285	293.9580751	2.128544166	1.089867025	5.46E-20	6.82E-17	Up
**Arl11**	15.97784788	10.30878518	23.06417625	2.231803441	1.158209972	0.001083502	0.034240213	Up
**Arx**	25.92598296	10.72835542	44.92301739	4.189425457	2.066752404	0.018176406	0.21772814	Up
**Ascl4**	3.835342448	1.999771073	6.129806666	3.050926272	1.609247317	0.044297404	0.354270181	Up
**Atp2a1**	85.16752166	42.49144849	138.5126131	3.259932908	1.704842273	0.026754681	0.270427062	Up
**BC035044**	12.04482865	8.110382926	16.9628858	2.09771101	1.068815939	0.00683257	0.122631412	Up
**BC147527**	2.640950069	1.025900411	4.659762142	4.59198382	2.199117559	0.029226705	0.282408907	Up
**Brs3**	9.189987363	5.264526511	14.09681343	2.685165537	1.425011031	0.035475368	0.311137364	Up
**Bst2**	83.55419925	54.7188028	119.5984448	2.186577823	1.128674696	2.00E-10	6.14E-08	Up
**Bsx**	17.47911111	5.549588555	32.3910143	5.849789781	2.548384781	0.032746406	0.298484775	Up
**C1qa**	837.5723397	509.4794662	1247.688432	2.449156475	1.29228495	1.53E-31	6.23E-28	Up
**C1qb**	1102.316218	717.4991722	1583.337526	2.20680729	1.141960651	5.41E-21	9.28E-18	Up
**C1qc**	977.0629049	617.3013865	1426.764803	2.311342287	1.208730924	1.07E-25	2.49E-22	Up
**Capn3**	161.9457733	111.8119366	224.6130692	2.010036598	1.00722177	5.16E-05	0.003224042	Up
**Ccdc170**	41.23011565	28.44047141	57.21717096	2.011723351	1.008431922	0.029942299	0.28491667	Up
**Ccdc180**	41.55861172	27.41364095	59.23982519	2.160320194	1.111245159	0.007998509	0.133799974	Up
**Ccdc78**	15.55503749	9.483030208	23.1450466	2.439237136	1.28643002	0.003387938	0.07854897	Up
**Ccl12**	13.56900057	8.020009509	20.5052394	2.543473026	1.346799794	0.00132054	0.039795049	Up
**Ccl3**	4.629600889	1.832839271	8.125552912	4.456698902	2.155975495	0.00302225	0.072615255	Up
**Ccl4**	2.72441325	0.991675987	4.890334828	4.886414	2.288776101	0.041392428	0.340768982	Up
**Ccl5**	6.610932415	4.228862725	9.588519527	2.264339327	1.179090172	0.028070263	0.276942574	Up
**Ccl9**	54.19708111	36.42788042	76.40858197	2.094653137	1.066711361	0.000122069	0.00630276	Up
**Ccr5**	90.62595203	61.74930466	126.7217612	2.053861845	1.038339141	2.04E-05	0.00150245	Up
**Cd180**	32.95448912	15.55674265	54.70167222	3.518503993	1.81496215	6.96E-09	1.49E-06	Up
**Cd22**	14.55943419	5.066039284	26.42617782	5.221138913	2.384364543	2.83E-08	5.29E-06	Up
**Cd300c2**	63.50812042	41.47977608	91.04355086	2.195170499	1.134332998	2.41E-10	6.88E-08	Up
**Cd33**	131.7802003	89.86127398	184.1788582	2.050534153	1.035999773	3.91E-14	2.89E-11	Up

**Cd48**	22.7626764	13.75788246	34.01866882	2.473719568	1.306681959	1.39E-05	0.001090149	Up
**Cd52**	36.78986238	15.16764106	63.81763903	4.207974859	2.073126085	3.89E-12	2.04E-09	Up
**Cd72**	6.481945046	3.793671023	9.842287574	2.579059502	1.366845058	0.029076692	0.281602766	Up
**Cd84**	53.46330757	31.70313295	80.66352584	2.541780285	1.345839327	8.06E-12	3.74E-09	Up
**Cd86**	50.49169259	31.96441795	73.6507859	2.305718657	1.205216487	2.38E-09	5.61E-07	Up
**Chrnb4**	81.58158706	42.53911783	130.3846736	3.066604897	1.616642301	0.00164731	0.046534373	Up
**Clec14a**	76.42270991	52.88928201	105.8394948	2.004540002	1.003271208	1.48E-06	0.00016265	Up
**Clec18a**	41.14678299	27.29925161	58.45619722	2.143247479	1.099798447	0.019347925	0.225605419	Up
**Clec5a**	39.45637518	23.97091976	58.81319445	2.450517226	1.293086288	2.29E-06	0.000239362	Up
**Clec7a**	18.38147218	6.089887003	33.74595365	5.554903172	2.473761764	8.26E-09	1.72E-06	Up
**Cnr2**	11.86925098	6.069663204	19.1187357	3.155486027	1.657862235	0.000658469	0.024034855	Up
**Cox6a2**	38.54343059	16.18522154	66.49119191	4.110020483	2.039145584	0.005005926	0.100446463	Up
**Cpn1**	5.185158342	2.85985785	8.091783958	2.849892821	1.510907663	0.029735019	0.284146042	Up
**Csf3r**	253.5293172	149.156775	383.994995	2.577389125	1.365910367	6.24E-34	3.38E-30	Up
**Cst7**	10.54427222	2.433555894	20.68266763	8.497636398	3.087061614	1.23E-06	0.000139258	Up
**Ctss**	1953.1271	1207.354471	2885.342886	2.389738525	1.256852773	3.45E-29	1.12E-25	Up
**Cuzd1**	2.16745973	0.404006427	4.371776358	10.58237784	3.40359193	0.017894688	0.215029131	Up
**Cwh43**	11.51219065	6.894264246	17.28459865	2.514543478	1.330296499	0.02795206	0.276942574	Up
**Cxcl10**	16.91169796	5.643757344	30.99662373	5.477663602	2.453560669	7.89E-07	9.56E-05	Up
**Cxcl13**	9.126878092	5.862511733	13.20733604	2.257127174	1.174487707	0.038350468	0.326310447	Up
**Cxcl9**	2.428658231	0.80573995	4.457306083	5.514934047	2.463343633	0.04690843	0.364039	Up
**Cxcr6**	1.977512455	0.606009641	3.691890973	6.089075043	2.606223093	0.046093632	0.36032161	Up
**Cyp2j13**	9.848799894	2.850383187	18.59682078	6.539937205	2.709276783	0.01599712	0.202065832	Up
**Ddx60**	32.05093848	17.38862307	50.37883274	2.896147289	1.534134975	1.08E-05	0.000876222	Up
**Dlx1**	56.4397967	15.63186475	107.4497116	6.879438296	2.782290774	1.93E-05	0.001436428	Up
**Dlx2**	11.71603605	3.865717409	21.52893435	5.58150032	2.480652974	0.012461556	0.173298851	Up
**Dlx5**	5.412628195	1.408961776	10.41721122	7.390508717	2.885673674	0.016785098	0.207331061	Up
**Dlx6**	10.3454289	3.662702602	18.69883678	5.122932343	2.356969839	0.003167294	0.074885523	Up
**Dok1**	19.48235588	12.49957814	28.21082805	2.256897685	1.174341017	0.003407727	0.0786755	Up
**Esyt3**	63.01408122	39.42481637	92.50066228	2.347783657	1.231299473	0.005512482	0.106283444	Up
**F9**	4.964648196	2.8195932	7.645966942	2.704249309	1.435228162	0.021194908	0.238909705	Up
**Fam167b**	3.180712793	0.809255937	6.145033862	7.601149728	2.926217653	0.004372264	0.091874114	Up
**Fam177a**	3.756583064	2.029713265	5.915170313	2.921732543	1.546824119	0.036605187	0.316243012	Up
**Fam83a**	1.418460764	0.40948757	2.679677257	6.493827149	2.699068984	0.049348013	0.375262067	Up
**Fcgr1**	74.6854448	48.47845002	107.4441883	2.216961847	1.148583942	1.34E-09	3.34E-07	Up
**Fcgr4**	7.433844155	4.038129545	11.67848742	2.881580879	1.526860513	0.005009028	0.100446463	Up
**Fezf1**	5.789079123	0	13.02542803	70.05778839	6.13047354	0.000122475	0.00630276	Up

**Gabra4**	1213.787761	800.5898053	1730.285205	2.161322064	1.111914068	0.032318726	0.295748209	Up
**Gal**	73.78851413	42.43414361	112.9814773	2.664747558	1.413998867	0.003125232	0.074106776	Up
**Gbp8**	4.44700363	2.449811196	6.943494172	2.845273862	1.508567521	0.02870229	0.279573567	Up
**Gcm2**	2.997793129	0	6.745034541	36.27098647	5.180744078	0.024364736	0.255935002	Up
**Gimap3**	6.092021112	3.437431958	9.410257554	2.732923528	1.450445091	0.012952756	0.176798459	Up
**Glrp1**	1.427091973	0.406467456	2.702872618	6.534322005	2.708037551	0.043887677	0.352381383	Up
**Gm17660**	8.735397269	5.251039987	13.09084387	2.4895351	1.315876356	0.006282643	0.11504958	Up
**Gm4951**	17.75303084	10.74374458	26.51463867	2.467124055	1.302830261	0.00026937	0.011889629	Up
**Gm5741**	22.01250879	1.583232493	47.54910417	29.90093892	4.902118882	0.001925265	0.05177496	Up
**Gm8773**	7.160225691	2.436532643	13.064842	5.37651175	2.426670464	0.017160299	0.210048781	Up
**Gna15**	35.91787981	24.74629272	49.88236367	2.021282465	1.015270946	6.04E-05	0.003641567	Up
**Gpr151**	62.91880058	10.37371205	128.6001612	12.40194703	3.632494728	0.00283529	0.068947231	Up
**Gpr183**	24.31105947	16.19890218	34.45125609	2.128655904	1.089942758	0.004146501	0.089082997	Up
**Gpr50**	44.59699377	24.84034129	69.29280938	2.793492925	1.482070166	0.007938612	0.133347343	Up
**Gpr84**	21.3017047	6.706858119	39.54526293	5.921929618	2.566067344	9.11E-12	4.11E-09	Up
**Gsx1**	5.263868004	2.839923572	8.293798543	2.933851758	1.552795976	0.029738858	0.284146042	Up
**H2-Oa**	9.83305308	5.055032827	15.8055784	3.124999892	1.64385614	0.001562752	0.044847678	Up
**H2-Ob**	18.21765604	9.053852091	29.67241097	3.263732703	1.706522907	1.30E-06	0.000145974	Up
**H2-Q9**	1.420843205	0.398010374	2.699384244	6.530096164	2.707104237	0.035778057	0.312442459	Up
**H2ac19**	11.50204473	7.48294459	16.5259199	2.20844618	1.143031674	0.003601019	0.081920656	Up
**Haao**	6.866899854	3.442354016	11.14758215	3.235046421	1.693786414	0.001432892	0.042011671	Up
**Has2**	4.059164211	1.617456917	7.111298329	4.403218302	2.138558372	0.007145255	0.125971065	Up
**Havcr2**	94.26521218	62.22275999	134.3182774	2.159032848	1.110385193	9.92E-10	2.56E-07	Up
**Hcar2**	5.920344757	1.01669612	12.04990555	11.91081686	3.574200453	1.48E-05	0.001146591	Up
**Hcrt**	137.7077233	2.636162365	306.5471744	116.3449762	6.862265106	1.29E-07	2.02E-05	Up
**Hdc**	161.0547314	106.4476739	229.3135532	2.15484001	1.107580758	0.00691542	0.123323549	Up
**Hes5**	71.61748296	47.86242239	101.3113087	2.117590666	1.082423741	0.0014704	0.042725774	Up
**Hhex**	22.61824426	12.73432345	34.97314527	2.749703028	1.459275814	0.000607194	0.022364292	Up
**Hk2**	124.4851481	79.94252662	180.163425	2.254282869	1.172668558	3.08E-09	6.84E-07	Up
**Hlx**	13.82644037	9.513631262	19.21745175	2.021643934	1.015528922	0.010676214	0.157928385	Up
**Hmcn2**	42.64213537	25.54872234	64.00890167	2.509124906	1.327184291	0.000768619	0.026619792	Up
**Hmx3**	10.76833108	5.466837474	17.3951981	3.191128406	1.674066662	0.000299982	0.012959072	Up
**Htr5b**	31.39340477	17.24362182	49.08063345	2.852785648	1.51237135	0.000134584	0.006811933	Up
**Hvcn1**	56.77875005	33.51373457	85.86001939	2.559336351	1.355769761	1.24E-10	4.04E-08	Up
**Ifi204**	25.68771415	15.27388048	38.70500623	2.540232241	1.344960401	0.000723686	0.025553988	Up
**Ifi206**	5.217336662	3.063619057	7.909483668	2.593255154	1.374764162	0.048871779	0.373663007	Up
**Ifi209**	15.45356567	8.283762482	24.41581966	2.94575636	1.558638112	0.000147948	0.007238302	Up

**Ifi27l2a**	36.0490536	20.47896832	55.51166019	2.713839709	1.440335512	1.50E-07	2.29E-05	Up
**Ifi44**	34.4021227	21.39356319	50.66282209	2.363287609	1.240795214	0.000176628	0.008488094	Up
**Ifit1**	109.2377265	75.09554892	151.9154485	2.020908754	1.015004184	8.42E-06	0.000710315	Up
**Ifit3**	247.3697903	131.4288018	392.2960258	2.984322334	1.577403368	0.004439094	0.092766329	Up
**Ifit3b**	140.2105179	72.74313078	224.5447519	3.085125842	1.625329338	8.13E-05	0.004572101	Up
**Igsf6**	25.5060157	16.95912415	36.18963014	2.133351797	1.093121891	6.38E-05	0.003780415	Up
**Il1a**	15.99021902	7.658728761	26.40458185	3.438588872	1.781816633	5.82E-07	7.33E-05	Up
**Il21r**	27.5409946	17.99325464	39.47566954	2.196367218	1.135119284	0.001240075	0.038012335	Up
**Irf8**	98.47611127	58.80311447	148.0673573	2.522101046	1.334626077	4.36E-13	2.62E-10	Up
**Isl1**	25.49936015	10.82898333	43.83733116	4.055192151	2.019770277	0.017062029	0.209201412	Up
**Itgax**	19.27771235	3.655275341	38.80575861	10.65300014	3.41318788	1.15E-09	2.93E-07	Up
**Klk1**	1.823580483	0	4.103056087	22.08473471	4.464977597	0.021024619	0.238147062	Up
**Kmo**	9.12790037	3.64607105	15.98018702	4.392406348	2.135011526	0.010685421	0.157928385	Up
**Krt12**	16.72497314	7.515184013	28.23720954	3.765254211	1.912747272	0.004249821	0.089882614	Up
**Lag3**	208.5936976	47.77118865	409.6218337	8.574888002	3.100117828	3.10E-79	2.52E-75	Up
**Lamb3**	18.69971288	12.35048526	26.6362474	2.157321544	1.109241223	0.028062863	0.276942574	Up
**Lat2**	42.72516594	29.36509804	59.42525081	2.025398343	1.018205677	3.90E-06	0.000376484	Up
**Ldlrad2**	9.524473548	4.218811447	16.15655118	3.824288567	1.935191388	0.010982501	0.159560605	Up
**Lhx6**	25.53053561	14.17259385	39.72796281	2.807353266	1.489210618	0.002381105	0.060621135	Up
**Lilra5**	6.545319912	3.876654557	9.881151605	2.559448214	1.355832816	0.026448434	0.269174132	Up
**Liph**	18.30427499	9.50079136	29.30862952	3.08544857	1.625480247	3.14E-06	0.000318796	Up
**Loxhd1**	12.86799592	5.468382206	22.11751307	4.049996857	2.017920788	0.011733119	0.165909404	Up
**Lrrc43**	26.56667381	15.77765797	40.0529436	2.539473623	1.344529489	0.009584206	0.148546051	Up
**Ly6i**	1.868262917	0.396252381	3.708276088	8.947587594	3.161498762	0.008572588	0.139383931	Up
**Ly86**	225.5603692	127.1112318	348.6217909	2.741671878	1.45505592	3.02E-20	4.09E-17	Up
**Ly9**	23.04065114	10.1254983	39.1845922	3.873226607	1.95353591	8.98E-08	1.46E-05	Up
**Mb**	2.728756999	0.794262755	5.146874805	6.40707059	2.679664885	0.01066109	0.157856054	Up
**Mbnl3**	4.375272092	2.421934748	6.816943772	2.823070063	1.497264934	0.041053235	0.339352515	Up
**Milr1**	6.739621653	2.243381798	12.35992147	5.536068721	2.468861852	0.000250142	0.011155264	Up
**Mpeg1**	712.7585841	483.4127304	999.4409013	2.067427192	1.047836523	7.35E-13	4.09E-10	Up
**Mpl**	1.21121609	0.194249167	2.482424743	8.926504667	3.158095373	0.025354467	0.262648343	Up
**Myct1**	8.710453071	4.68393229	13.74360405	2.9472447	1.559366848	0.008623093	0.139785326	Up
**Mymk**	7.954112738	4.642444402	12.09369816	2.601618367	1.379409348	0.012598992	0.174463276	Up
**Myo1f**	84.01636905	53.19880268	122.538327	2.304985244	1.204757515	3.41E-11	1.42E-08	Up
**Naalad2**	58.68337371	36.15274036	86.8466654	2.402345754	1.264443804	6.03E-10	1.61E-07	Up
**Naip2**	45.25429351	30.3417069	63.89502678	2.105880333	1.074423457	1.97E-07	2.91E-05	Up
**Ncf4**	21.90738706	14.9367498	30.62068362	2.047409331	1.033799564	0.002584588	0.064255858	Up

**Nek5**	17.22517964	10.80773424	25.24698639	2.331496979	1.221256561	0.026275164	0.268082596	Up
**Neurl3**	22.26559716	14.31062536	32.20931191	2.24906106	1.169322829	0.000284523	0.012423415	Up
**Nkx3-1**	16.97086223	7.089153239	29.32299846	4.141566326	2.050176493	0.008441924	0.137949872	Up
**Nlrc5**	68.13922323	43.17671241	99.34236175	2.296881825	1.199676632	2.54E-07	3.62E-05	Up
**Nlrp3**	28.01316201	19.01358093	39.26263836	2.064424	1.045739308	0.00052203	0.019982912	Up
**Notum**	62.57034462	40.17260785	90.56751559	2.257730874	1.174873524	0.000250672	0.011155264	Up
**Npvf**	5.364594785	0.194249167	11.82752681	42.63916313	5.414107218	0.001290087	0.039167999	Up
**Nr5a1**	4.710923243	0.194249167	10.35676584	37.3316615	5.222327814	0.007565528	0.129599258	Up
**Oasl1**	11.48514292	6.441907045	17.78918776	2.752781286	1.46088999	0.001310942	0.039579253	Up
**Oasl2**	202.7732244	120.8794337	305.1404628	2.523994733	1.3357089	3.51E-16	3.17E-13	Up
**Otoa**	4.517521756	2.224872629	7.383333164	3.320330696	1.731326937	0.031036156	0.290894569	Up
**P2ry13**	179.423159	112.7889475	262.7159234	2.331221167	1.221085882	1.23E-13	8.32E-11	Up
**Pdcd1**	5.176760953	1.017751078	10.3755233	10.24050053	3.356214327	6.87E-05	0.004015603	Up
**Pifo**	10.88060769	6.638372702	16.18340141	2.435672435	1.284320123	0.015121558	0.194936086	Up
**Pik3cg**	93.03503243	61.62517377	132.2973557	2.146510156	1.101992999	2.74E-10	7.67E-08	Up
**Pilra**	6.331367367	4.068837982	9.159529099	2.255656715	1.173547523	0.038646444	0.328141242	Up
**Pld4**	243.050259	166.018576	339.3398628	2.044968187	1.0320784	7.56E-13	4.09E-10	Up
**Pmch**	731.8110255	22.48136341	1618.473103	71.99405516	6.169805877	2.36E-07	3.39E-05	Up
**Pomc**	141.1231052	28.98821077	281.2917232	9.704844416	3.278705084	0.001334333	0.03998815	Up
**Ppp1r3a**	9.819151911	2.026448612	19.56003103	9.664288293	3.272663492	0.008284922	0.136760151	Up
**Prdm13**	2.253153567	0	5.069595526	27.26361517	4.768904972	0.045980928	0.360110032	Up
**Prkcd**	3411.213114	1344.810067	5994.216923	4.457290103	2.156166862	0.005314196	0.104375443	Up
**Prkch**	223.1200905	152.081029	311.9189175	2.05153307	1.03670241	0.005429337	0.105615235	Up
**Prl**	751.0888336	120.9578372	1538.752579	12.72091118	3.669130108	0.023163696	0.24834846	Up
**Ptafr**	23.38334474	15.44255124	33.30933661	2.163993883	1.113696421	0.003428447	0.078990453	Up
**Ptpn18**	18.26679625	12.36110091	25.64891542	2.077150151	1.054605508	0.002522214	0.063418447	Up
**Ptpn6**	99.48604933	50.06911069	161.2572226	3.225223777	1.689399263	7.11E-17	6.79E-14	Up
**Ptprc**	108.0772592	71.45785237	153.8515178	2.154549059	1.107385949	1.58E-09	3.82E-07	Up
**Pvrig**	4.427924999	2.434159152	6.920132308	2.845898149	1.508884031	0.046500277	0.362603937	Up
**Pycr1**	43.59460544	30.04787851	60.5280141	2.01523027	1.010944697	0.021183917	0.238909705	Up
**Ramp3**	416.7126869	184.1857416	707.3713686	3.840625973	1.941341471	0.011549108	0.16426634	Up
**Rasal3**	90.74151464	60.13275879	129.0024595	2.147007001	1.102326896	7.83E-09	1.65E-06	Up
**Rasgrp1**	2735.220713	1734.314564	3986.353399	2.298540021	1.200717786	0.036541559	0.315883203	Up
**Rgs14**	58.43208376	38.18013934	83.74701428	2.195204243	1.134355175	0.040520235	0.337350171	Up
**Rgs9bp**	10.78870775	5.044861924	17.96851504	3.566443572	1.834486147	0.00576617	0.109619241	Up
**Samsn1**	23.0981496	13.12817122	35.56062258	2.704918404	1.435585075	6.58E-07	8.10E-05	Up
**Satb2**	25.18401475	16.44657508	36.10581435	2.196446712	1.135171498	0.02449452	0.257018402	Up

**Scube2**	90.47852117	51.54031487	139.151279	2.700841882	1.433409181	0.004503885	0.093311989	Up
**Sh2d1b1**	2.091238919	0.602474617	3.952194296	6.519313704	2.704720098	0.014423672	0.189090958	Up
**Sh3rf2**	2.290690229	0.808200979	4.143801791	5.138733978	2.361412969	0.040588241	0.337586761	Up
**Siglecf**	13.4638111	7.038428973	21.49553876	3.04189487	1.604970293	0.000112713	0.005963526	Up
**Slamf6**	5.408885048	2.237592907	9.373000225	4.205337871	2.072221716	0.001663825	0.046595702	Up
**Slamf8**	7.911145624	4.228511611	12.51443814	2.956747508	1.56401105	0.006271522	0.114975544	Up
**Slamf9**	20.95611233	9.692353299	35.03581111	3.610768089	1.852305762	7.44E-08	1.23E-05	Up
**Slc15a3**	34.72217119	20.41653375	52.604218	2.57470698	1.364408253	1.54E-07	2.34E-05	Up
**Slfn8**	43.57646371	26.28786754	65.18720891	2.481004318	1.310924246	8.97E-07	0.000106372	Up
**Slfn9**	21.04161361	13.16055011	30.89294299	2.347111591	1.230886435	0.000105663	0.005670954	Up
**Smim43**	11.07148681	3.64131808	20.35919772	5.587748761	2.482267155	0.000909979	0.030396242	Up
**Smpd5**	7.606066361	5.075255816	10.76957954	2.131374036	1.091783795	0.0489767	0.373663007	Up
**Snx31**	14.84204457	4.441828223	27.842315	6.270626409	2.648609569	0.004968857	0.100135418	Up
**Sp8**	7.060726916	2.417407976	12.86487559	5.322923544	2.412218844	0.006237423	0.114738922	Up
**Spint1**	33.77810606	20.42904768	50.46442904	2.47210198	1.305738259	2.00E-07	2.93E-05	Up
**Sspo**	424.3087833	233.7502954	662.5068933	2.834359159	1.503022583	0.01074307	0.158204612	Up
**Stum**	1715.177163	1136.586567	2438.415407	2.145427446	1.101265113	0.030490373	0.287771723	Up
**Susd3**	24.27496128	15.21060502	35.6054066	2.342278819	1.227912821	0.000334462	0.014258968	Up
**Synpo2**	831.0133489	436.4377194	1324.232886	3.034226824	1.601328938	0.032278127	0.295748209	Up
**Syt15**	31.97355728	21.86844142	44.60495211	2.043364271	1.030946417	0.012708683	0.175235262	Up
**Tac2**	122.570547	53.07133171	209.4445661	3.947438981	1.980916965	0.017667056	0.213675351	Up
**Tbx19**	4.000693574	0.808200979	7.991309318	9.906292853	3.308345271	0.003895279	0.085849414	Up
**Tbx3**	92.48083369	61.45067814	131.2685281	2.139417546	1.097218078	0.000133089	0.006776689	Up
**Tbxa2r**	11.7056748	8.052852189	16.27170307	2.015835002	1.011377558	0.015306768	0.196958648	Up
**Tcte1**	22.88387454	15.29762453	32.36668705	2.114962135	1.080631834	0.019244289	0.225282118	Up
**Tent5c**	78.57435798	52.15104011	111.6035053	2.140084989	1.097668092	1.34E-08	2.65E-06	Up
**Tlr1**	15.35632467	10.10163371	21.92468838	2.170160562	1.117801786	0.002380289	0.060621135	Up
**Tlr13**	69.9956939	47.48911	98.12892378	2.064068082	1.045490558	1.20E-08	2.40E-06	Up
**Tlr9**	37.41841425	23.67214822	54.60124677	2.308594064	1.207014516	1.38E-05	0.001086063	Up
**Tmco5**	2.601521028	0.601771581	5.101207836	8.453916351	3.079619837	0.017440141	0.21203608	Up
**Tnfaip8l2**	31.21378441	21.02880993	43.94500251	2.089017655	1.062824685	0.000710329	0.025246999	Up
**Tns4**	6.692586041	4.237878891	9.760969978	2.308080621	1.206693618	0.048823217	0.373544751	Up
**Traf3ip3**	14.62529594	9.949902249	20.46953805	2.061418001	1.043637075	0.009972074	0.152090521	Up
**Trem2**	199.401714	112.3543879	308.2108716	2.743709921	1.456127961	1.21E-18	1.41E-15	Up
**Treml2**	10.70412582	5.266592253	17.50104279	3.327477307	1.734428827	5.58E-05	0.003422936	Up
**Trh**	79.21396605	53.97723827	110.7598758	2.053868773	1.038344007	0.010136029	0.153488091	Up
**Trim40**	11.65377197	3.791686831	21.4813784	5.656316524	2.499862855	0.026380369	0.268649737	Up

**Tubb1**	3.658558231	0.813682122	7.214653368	8.885160714	3.151397872	0.001191074	0.036780635	Up
**Tyrobp**	248.6661408	157.192651	363.008003	2.309424697	1.207533505	3.60E-11	1.45E-08	Up
**Uba7**	100.3525538	68.30373307	140.4135798	2.058685608	1.041723526	1.93E-11	8.27E-09	Up
**Ugt1a7c**	5.394951786	2.656592517	8.817900872	3.346191492	1.742520009	0.010825548	0.158649521	Up
**Umodl1**	9.495367554	4.469969057	15.77711568	3.540306539	1.823874282	0.020487417	0.234515237	Up
**Usp18**	60.49640937	41.35553188	84.42250623	2.042841277	1.030577115	6.70E-06	0.000582063	Up
**Wnt9a**	65.21585334	36.13161434	101.5711521	2.813773313	1.492506105	0.001456511	0.042550553	Up
**Wnt9b**	66.06804766	28.40941483	113.1413387	3.982797977	1.993782302	0.001689602	0.047221447	Up
**Zbp1**	11.46597573	6.426888727	17.76483448	2.756076879	1.462616132	0.00874298	0.140841876	Up
**2210418O10Ril**	19.68554405	29.71907762	7.143627084	0.240579029	-2.055417202	0.002580884	0.064255858	Down
**Acod1**	1.449742353	2.41010563	0.249288256	0.123934321	-3.012352328	0.033289296	0.300900406	Down
**Bcl3**	42.19785195	60.35710432	19.49878648	0.322685139	-1.631800957	3.00E-07	4.06E-05	Down
**Bfsp2**	79.9860526	109.1281195	43.55846899	0.399441326	-1.323944494	0.014324411	0.188245473	Down
**Bub1**	3.573051478	5.453602285	1.22236297	0.225555255	-2.148447199	0.01272649	0.175331963	Down
**Catspere1**	8.52284511	11.143369	5.247190251	0.467633434	-1.096550015	0.030335439	0.287067192	Down
**Cbl**	1418.923992	2049.033132	631.2875663	0.308050421	-1.698761586	2.19E-128	3.55E-124	Down
**Cebpd**	153.6615424	213.038258	79.44064795	0.372623972	-1.424207605	2.68E-08	5.06E-06	Down
**Ch25h**	50.8794533	74.71246699	21.08818619	0.281664626	-1.827949704	0.002353648	0.060288662	Down
**Clec4e**	1.684535228	3.032163411	0	0.059874106	-4.06192399	0.002170341	0.056951292	Down
**Csf3**	6.369841678	11.27359113	0.240154862	0.026551481	-5.235063847	0.001345314	0.040242975	Down
**Cxcl1**	19.49437147	33.11517843	2.468362775	0.074518861	-3.746250569	6.44E-06	0.000570191	Down
**Dsc3**	10.72196178	14.33328344	6.207809699	0.431181849	-1.213631647	0.010217163	0.15409228	Down
**Epyc**	10.59136557	15.69667163	4.209732998	0.267529178	-1.90223185	0.004023682	0.087492187	Down
**F10**	1.995315255	3.386688656	0.256098503	0.088068473	-3.505230535	0.012793714	0.175691265	Down
**Gabrr1**	17.22668746	22.86856468	10.17434093	0.444106578	-1.171022155	0.0213026	0.239293317	Down
**Glyat**	9.406474631	12.36205609	5.711997808	0.460660492	-1.118224224	0.024139682	0.254280715	Down
**Gm2956**	4.813165198	6.693523152	2.462717756	0.368841693	-1.438926353	0.030910167	0.289880967	Down
**Gm8094**	6.151547102	8.49621307	3.220714643	0.378034681	-1.403409502	0.01750586	0.212651572	Down
**Gm9237**	2.673191604	4.040238652	0.964382794	0.242389063	-2.044603491	0.048073011	0.36937082	Down
**Gprc5a**	12.55406579	17.81358352	5.979668629	0.334396959	-1.580366369	0.006860249	0.122992295	Down
**Grrp1**	165.1699331	216.3757306	101.1626861	0.46732728	-1.097494838	2.82E-09	6.35E-07	Down
**Icam1**	149.7520837	207.894458	77.0741158	0.370793191	-1.431313344	0.00014518	0.007129734	Down
**Il31ra**	19.33223396	26.85429546	9.929657083	0.37075899	-1.431446419	0.039497873	0.332121722	Down
**Itih4**	6.134457228	8.470546315	3.21434587	0.378673818	-1.400972421	0.03485729	0.308129287	Down
**Lcn2**	265.3385411	426.2871457	64.15278543	0.150430483	-2.732831157	7.51E-05	0.004340997	Down
**Maff**	74.42959694	97.57331215	45.49995292	0.465279219	-1.103831342	4.22E-05	0.002772847	Down
**Marveld3**	12.26727554	15.99528044	7.607269414	0.477389349	-1.066761716	0.009222033	0.145430558	Down

**Mcoln2**	1.878290737	2.995170209	0.482191397	0.163647118	-2.611339903	0.047920792	0.368899255	Down
**Mmp8**	3.322160283	5.370700301	0.761485261	0.138912767	-2.847748897	0.021742003	0.241721069	Down
**Mpz**	14.87859086	21.29128233	6.862726519	0.322838159	-1.631116983	0.035198933	0.310052207	Down
**Mpzl2**	66.17366785	86.85062608	40.32747005	0.465051413	-1.104537876	0.001602173	0.045576346	Down
**Plaur**	43.9025484	64.32005792	18.3806615	0.284518756	-1.813404333	9.20E-11	3.25E-08	Down
**Plscr5**	10.85243863	14.39909603	6.419116872	0.446075427	-1.16464042	0.013690775	0.18302819	Down
**Pou4f2**	107.3835371	139.7775342	66.89104058	0.478246125	-1.064174814	0.031258208	0.291796013	Down
**Ptgs2**	139.0236192	188.7119856	76.91316119	0.407767728	-1.294180495	0.002608627	0.064591365	Down
**Rec114**	11.54297038	15.21537784	6.952461063	0.456089969	-1.132609653	0.039053078	0.330569089	Down
**Rxfp2**	28.24512316	36.46362017	17.97200189	0.493232721	-1.019659583	0.021229667	0.239135568	Down
**Sele**	12.5068623	21.3288766	1.47934442	0.069367575	-3.849594735	0.000266491	0.011794582	Down
**Selp**	37.39757307	65.55219465	2.204296107	0.033715335	-4.890451269	6.26E-08	1.07E-05	Down
**Serpina3f**	52.07247087	90.15313315	4.471643022	0.049437261	-4.338257372	9.52E-12	4.18E-09	Down
**Serpina3g**	110.7365794	147.1065613	65.27410189	0.443123057	-1.174220698	0.000923747	0.030621285	Down
**Serpina3i**	4.368108066	6.26391318	1.998351673	0.316233042	-1.660939979	0.02500221	0.259994316	Down
**Slc6a20b**	3.898030427	6.427328186	0.736408227	0.114801375	-3.122788174	0.004332864	0.091282381	Down
**Socs3**	73.50179769	96.42799788	44.84404745	0.46498535	-1.104742831	0.004469984	0.093084551	Down
**Spon2**	8.154852473	10.94605554	4.665848635	0.427824218	-1.224909943	0.019818433	0.229512617	Down
**Steap4**	37.14065558	52.55658344	17.87074575	0.340114958	-1.55590564	0.010504051	0.156386162	Down
**Timp1**	15.37569244	21.94442697	7.16477427	0.326054195	-1.616816313	0.003047566	0.072796491	Down
**Tmem252**	248.0597739	400.5122668	57.49415778	0.143436174	-2.801519183	1.14E-07	1.81E-05	Down
