# Supplementary figures and images for: The E3 ligase c-Cbl modulates microglial phenotypes and contributes to Parkinson’s disease pathology
Source: Cell Death Discov. 2025 Apr 17;11:184. doi: 10.1038/s41420-025-02482-0 (PMC12006326; doi:10.1038/s41420-025-02482-0)

Figure 1A

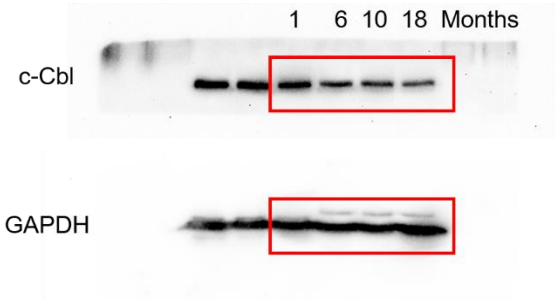

Figure 1C

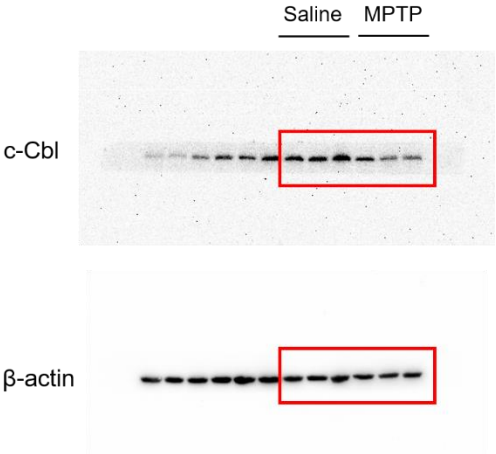

Figure 6H

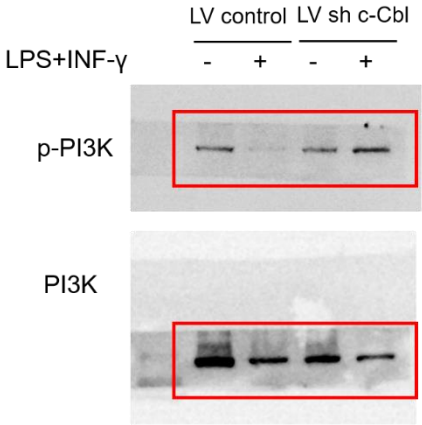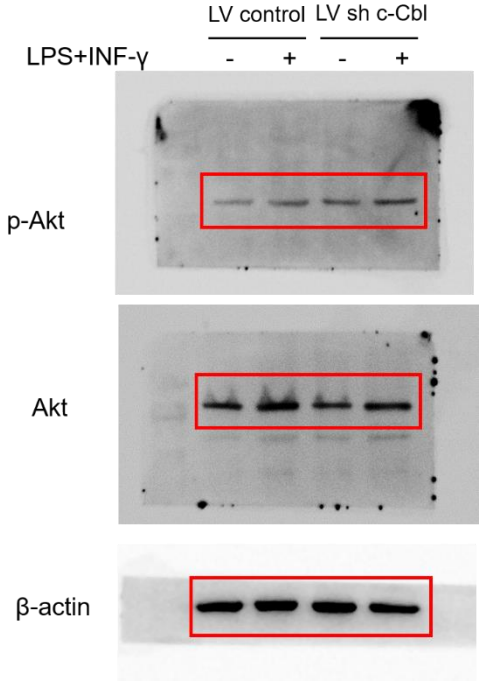

Supplement: Supplementary file 3 — The full length uncropped original western blots used in this manuscript. [file 41420_2025_2482_MOESM3_ESM.pdf]
